# Acupuncture as a Complementary Therapy for Cancer-Induced Bone Pain: A Systematic Review and Meta-Analysis

**DOI:** 10.3389/fpain.2022.925013

**Published:** 2022-08-01

**Authors:** Zhaobo Yan, Zhimiao MuRong, Bixiu Huo, Huan Zhong, Chun Yi, Mailan Liu, Mi Liu

**Affiliations:** ^1^College of Acupuncture and Moxibustion, Hunan University of Chinese Medicine, Hunan, China; ^2^Hospital of Chengdu University of Traditional Chinese Medicine, Chengdu, China; ^3^Department of Pathology, Hunan University of Chinese Medicine, Hunan, China

**Keywords:** acupuncture, cancer-induced bone pain, meta-analysis, cancer pain, a systematic review

## Abstract

**Background:**

Cancer-induced bone pain (CIBP) is a special type of cancer pain and lacks safe and effective treatments. Acupuncture is a potentially valuable treatment for CIBP, studies evaluating the effect of acupuncture on CIBP have increased significantly, but the safety and efficacy of acupuncture to control CIBP remains controversial.

**Objective:**

To provide the first meta-analysis to evaluate the safety and efficacy of acupuncture in CIBP management.

**Data Sources:**

CNKI, CBM, Wanfang, VIP Database, PubMed, Embase, and Cochrane Library were searched from their inception until 1 June 2022.

**Study Selection:**

RCTs with primary bone tumor patients or other types of primary cancer companied by bone metastases as the research subjects and to evaluate the efficacy of acupuncture treatment alone or combined with the control treatment were included. Meanwhile, RCTs should choose the pain score as the primary outcome and pain relief rate, frequency of breakthrough pain, analgesic onset time, analgesia duration, quality of life, and adverse events as reference outcomes.

**Data Collection and Analysis:**

We designed a data-extraction form that was used to extract key information from the articles. Data extraction study evaluation was conducted independently by two reviewers, and a third reviewer would resolve any disagreements. The risk of bias was assessed by the Cochrane Collaboration's tool for assessing the risk bias. The quality of the evidence for main outcomes was evaluated by the GRADE system. Mean differences (MD), relative risk (RR), and 95% confidence intervals (CIs) were calculated. The forest plots were performed using the Review Manager Software (5.3 version). Subgroup analysis was used to investigate the possible sources of potential heterogeneity. Descriptive analysis was performed in case of unacceptable clinical heterogeneity.

**Results:**

Thirteen RCTs (with 1,069 patients) were included, and all studies were at high risk of bias owing to lack of blinding or other bias. Eleven studies evaluated the effectiveness of acupuncture as a complementary therapy, and showed that acupuncture plus control treatment (compared with control treatment) was connected with reduced pain intensity (MD = −1.34, 95% CI −1.74 to −0.94; Q < 0.1; *I*^2^ = 98%, *P* < 0.01). Subgroup analyses based on acupoints type partly explain the potential heterogeneity. The results also showed that acupuncture plus control treatment (compared with control treatment) was connected with relieving pain intensity, increasing the pain relief rate, reducing the frequency of breakthrough pain, shortening analgesic onset time, extending the analgesic duration, and improving the quality of life. We have no sufficient evidence to prove the effectiveness of acupuncture alone. Four RCTs reported only adverse events related to opioids' side effects. Evidence was qualified as “very low” because of low methodological quality, considerable heterogeneity, or a low number of included studies.

**Conclusion:**

Acupuncture has a certain effect as a complementary therapy on pain management of CIBP, which not only mitigates the pain intensity but also improves the quality of life and reduces the incidence of opioids' side effects, although the evidence level was very low. In future, a larger sample size and rigorously designed RCTs are needed to provide sufficient evidence to identify the efficacy and safety of acupuncture as a treatment for CIBP.

## Introduction

Cancer pain is highly prevalent in patients with cancer (1). Uncontrollable pain is a primary factor, compromising the quality of life for cancer patients (2). The factors of cancer pain include not only the pathology associated with cancer itself (e.g., bone, soft tissue or visceral metastases) but also comorbidities caused by various cancer treatments (3), such as mucositis induced by chemotherapy (4), and musculoskeletal symptoms from hormone therapy (5). CIBP is a common source of moderate and severe cancer pain (6). When cancer invades bone and surrounding tissues, cancer cells can release pain mediators and cause peripherally and centrally neuropathic changes, which contribute to a mixed-mechanism pain state (7). Bone metastasis is the most contributor to CIBP, which is a common symptom in advanced cancers (8), with a prevalence of 70% in prostate and breast cancer patients and 30% in lung, bladder, and thyroid cancer patients (9), and ~70% of cancer patients with bone metastasis will experience CIBP (10). CIBP exists as a combination of background and breakthrough pain (7). Breakthrough pain is characterized by rapid onset and short duration, which limits the efficacy of standard analgesics in controlling CIBP (6). Despite this, oral morphine is still an important choice for patients who can receive oral medication, according to a three-step ladder protocol recommended by the World Health Organization (WHO) (11). In addition, other medications such as zoledronic acid (12), radiotherapy (6), nerve block (13), and radionuclide therapy (9) are also prescribed to alleviate CIBP. However, the abovementioned treatment methods cannot effectively control CIBP and produce unwelcome side effects (14–16), which result in a reduction in patient compliance and negatively impact patient activities of daily living. Hence, there is an urgent need to explore an effective treatment with fewer side effects to alleviate CIBP (17, 18).

Acupuncture is an ancient traditional Chinese medical practice involving stimulating specific acupoints and meridian channels to control pain and other symptoms (19). A variety of acupuncture techniques have been devised in clinical use, including manual acupuncture (20), auricular point acupressure (21), moxibustion (22), catgut-embedding therapy (23), and so on. The effect of acupuncture on pain-relieving has been accepted by leading organizations in the medical community, such as the American Society for Clinical Oncology (24) and the National Comprehensive Cancer Network (25), and acupuncture, as a non-pharmacological intervention, has been widely used for cancer pain management (26). Acupuncture for cancer pain has been a research hot spot in the field of cancer research. Many systematic reviews have established the association between acupuncture and cancer pain (3, 27, 28). However, no recent systematic reviews or meta-analyses have focused on the safety and efficacy of acupuncture for CIBP. A previous meta-analysis (3), including 29 RCTs, assessed the effectiveness of acupuncture for different types of cancer pain (malignancy-related pain, chemotherapy or radiation therapy-induced pain, surgery-induced pain, and hormone therapy-induced pain), but this systematic review did not specifically mention CIBP. Another meta-analysis (28) in 2019 includes only one RCT evaluating the effectiveness of acupuncture on CIBP; this result may not be sufficient to prove acupuncture's effectiveness on CIBP. Research on acupuncture for CIBP has continued in recent years, but the findings have been inconsistent. In addition, the use of acupuncture to control CIBP remains controversial, and there is a concern that acupuncture may increase tumor growth (29, 30). Therefore, this analysis aimed to evaluate the efficacy and safety of acupuncture in CIBP management based on current relevant RCTs to provide scientific references for future research and practice. This systematic review and meta-analysis have been checked with PRISMA checklist (see Supplementary Table 1).

## Methods

### Search Strategies

Four Chinese-language databases and three English-language databases were searched from their inception through 1 June 2022: China National Knowledge Infrastructure (CNKI), Chinese Biomedical Literature Database (CBM), Wanfang, VIP Database for Chinese Technical Periodicals, PubMed, Embase, and Cochrane Library. The restriction was set for the language (English and Chinese) but not for investigation regions. Reviewers independently searched the articles using the following search terms: (cancer-induced bone pain OR bone cancer pain OR bone metastasis pain OR bone cancer OR bone metastasis OR Bone neoplasm OR cancer of the bone) AND (acupuncture OR electroacupuncture OR manual acupuncture OR moxibustion OR catgut-embedding therapy OR transcutaneous electrical acupoint stimulation OR auricular point OR thumb-tack acupuncture OR wrist-ankle acupuncture OR warm acupuncture). The equivalent search words were used in the Chinese databases. Please refer to Supplementary Table 2 for a detailed description of the specific research strategy corresponding to each database. The EndNote software was used to manage citations obtained through the database search.

### Inclusion Criteria

The inclusion criteria were (1) study design: only randomized controlled trials (RCTs) were eligible, and (2) participants: patients were diagnosed with primary bone cancer or other types of primary cancer companied by bone metastases. CIBP patients were diagnosed by imaging or biopsy. There was well-defined localized pain in CIBP patients, and (3) the number of subjects in each group of one RCT should be greater than or equal to 20, and (4) intervention and control: the intervention group received at least one of the following acupuncture treatments: manual acupuncture, auricular point acupressure, moxibustion, catgut-embedding therapy, thumb-tack acupuncture, wrist-ankle acupuncture, transcutaneous electrical acupoint stimulation, warm acupuncture, and electroacupuncture with or without the combination of the control treatment, regardless of acupoints, frequency, and sessions. Treatments in the control group can be oral morphine, morphine injection, fentanyl, parenteral morphine, zoledronic acid, nerve block, sham acupuncture, placebo, or usual care, and (5) outcome measures: pain intensity was described as the primary outcome, which should be reported in each included study. Pain intensity can be measured by a pain measurement, such as the Visual Analog Scale for Pain (VAS Pain), the Numeric Rating Scale for Pain (NRS Pain), or the item of “the current pain item” in the Brief Pain Inventory (BPI). We determined pain relief rate, frequency of breakthrough pain, analgesic onset time, analgesia duration, quality of life, and adverse events as reference outcomes.

### Exclusion Criteria

The exclusion criteria were (1) studies related to qualitative studies, animal studies, case reports, or other topics not related to RCTs, (2) the same study, which was republished, (3) studies with incomplete original data, or data that could not be extracted and still unavailable after contacting the authors, and (4) there are obvious errors in the outcome, and the statistical methods are inappropriate, and (5) when experimental group applies a pain relief method which is different from the control group.

### Data Screening and Extraction

Two reviewers (Zhao-bo Yan and Zhi-miao MuRong) examined all studies independently according to the inclusion criteria. We designed a data-extraction form using Microsoft Excel 2016 that was used to extract key information from the articles. The key information will be extracted from studies that meet the inclusion criteria for data analysis. The extracted data mainly included the first author, publication year, country, sample size, age, cancer type, therapy types, control group types, outcomes, duration of intervention, and acupoints. If the above information was unclear, the report's corresponding authors were contacted to provide clarification or additional detail. The extracted data were cross-checked and discrepancies were resolved by discussion and mediated by a third reviewer (Mai-lan Liu).

### Evaluation of Risk of Bias and Quality of the Evidence

First, included studies' quality was appraised with the Cochrane collaboration tool (31) in seven terms, which included random sequence generation, allocation concealment, blinding of participants and personnel, blinding of outcome assessment, incomplete outcome data, selective reporting, and other bias. Each term would be rated as low risk, high risk, and unclear. The Grading of Recommendations Assessment, Development, and Evaluation System (GRADE) (32) would be used to grade the quality of the evidence for main outcomes, and the quality of the evidence was rated as high, moderate, low, or very low according to the GRADE grading scale. The evaluation was conducted independently by two reviewers (Zhao-bo Yan and Zhi-miao MuRong); any disagreements would be resolved by a third reviewer (Mai-lan Liu).

### Data Analysis

The meta-analysis was performed using the Review Manager Software (5.3 version). The effect size of dichotomous and continuous data was presented as relative risk (RR) and mean difference (MD); both were reported with a 95% confidence interval. The heterogeneity among trials was identified by the *I*^2^ test and quantified by the *I*^2^ statistic (33). When the heterogeneity test was acceptable (*P* > 0.1, *I*^2^ ≤ 50%), a fixed-effects model was used for meta-analysis. When the heterogeneity was significant (*P* ≤ 0.1, *I*^2^ > 50%), a random-effects model was used for meta-analysis. Subgroup analyses would be performed to analyze the source of heterogeneity. Descriptive analysis should be selected instead of a meta-analysis if *P* < 0.1 and the sources of diversity are unknown. Pooled effects were calculated, and a two-sided *P*-value < 0.05 was considered to indicate statistical significance.

## Results

### Literature Search

**Figure 1:**
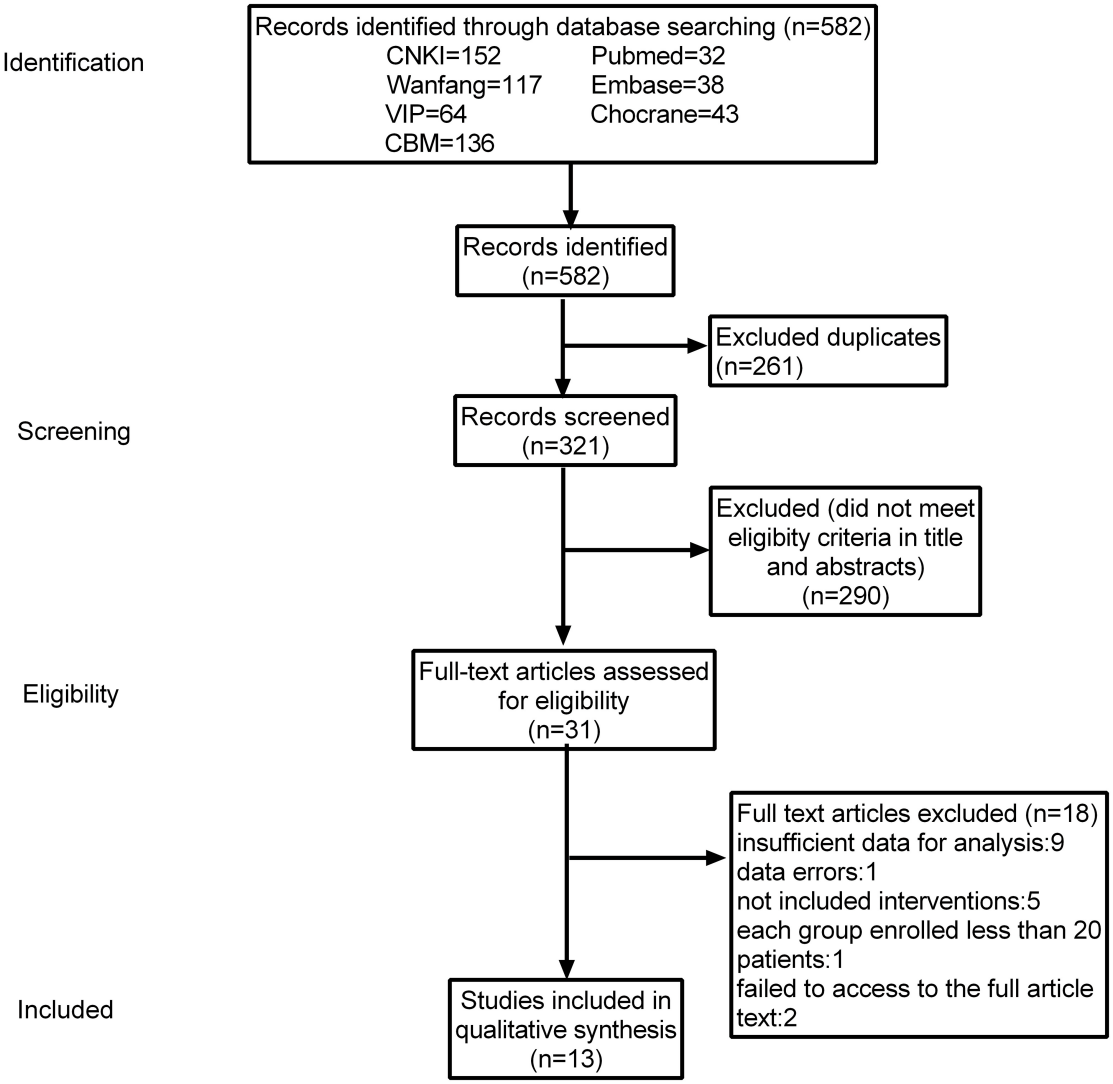
Literature search and screening process.

Through database searching, 582 papers were screened, from which 261 duplicate papers were removed. Further titles and abstracts reviewed excluded 290 records as they were animal studies, qualitative studies, case reports, or other topics unrelated to the present study. After a cautious reading of the full-text versions of the remaining 31 studies, 18 studies were excluded for not satisfying the inclusion criteria. Therefore, 13 studies were included, of which 12 (34–45) were published in Chinese, and only one study (46) was published in English but conducted in China. The process of trial screening and selection was present in Figure 1.

### Study Characteristics

We included 13 studies (34–46) in the final qualitative analysis, all published between 2015 and 2021. A total of 1,069 patients were included, with 536 in the experimental group and 533 in the control group. The largest sample size was 144 cases in a study by Yao et al. (44), while the smallest sample size was 48 cases in the study by Du et al. (34). The participants ranged from 18 to 84 years and included patients who were all diagnosed with bone metastases. Seven kinds of acupuncture techniques were applied across included studies, including wrist-ankle acupuncture (WAA), thumb-tack acupuncture (TTA), auricular point acupressure (APA), manual acupuncture (MA), warm acupuncture (WA), transcutaneous electrical acupoint stimulation (TEAS), and catgut-embedding therapy (CET). Control treatments in control groups comprised opioids, zoledronic acid, and nerve block. The needle retention time, the number of sessions, duration, and acupuncture point varied with acupuncture types; for details, see Table 1. Eleven studies (34–39, 41–45) compared acupuncture plus control treatment with control treatment, one study (40) compared wrist-ankle acupuncture treatment alone with analgesic therapy, and the remaining one (46) compared TEAS with the combination of analgesic therapy and sham TEAS. Pain intensity as a primary outcome was used to evaluate the effect of acupuncture; VAS and NRS were the most frequently used measurement tools for pain intensity. Five studies (36, 40, 44–46) reported VAS scores, seven studies (35, 37–39, 41–43) reported NRS scores, and in addition, one study (34) appraised the pain intensity by BPI scores. In terms of reference outcomes, eight studies (35, 37–39, 41–43, 45) reported a pain relief rate, one study (34) measured the changes in the frequency of breakthrough pain, and two studies (37, 39) measured the analgesic onset time and analgesia duration after acupuncture treatments. Eight studies (35–39, 41, 44, 45) focused on quality of life, and the methods used for quality of life varied widely. Among eight studies, three studies (37, 39, 44) used the European Organization for Research and Treatment of Cancer Quality of Life Questionnaire Core 30 (EORTCQLQ-C30) and three studies (35, 38, 41) chose the Karnofsky Performance Status Scale (KPS). In addition, the Eastern Cooperative Oncology Group (ECOG) and the Prostate Cancer-Specific Quality of Life Instrument (PROSQOLI) were used in the remaining two studies, respectively. Four studies (34, 35, 39, 44) reported adverse events. The specific characteristics of the included studies are shown in Table 2.

**Table 1:** The acupuncture techniques and corresponding retention times, number of sessions, and acupuncture prescript.

**Acupoint**	**Acupuncture techniques**	**References**	**Acupuncture prescription**	**Needle retention time, sessions, and duration**
Wrist-ankle acupoints	WAA	Liu et al. (37)	According to WAA theory, select the treatment area on the wrist or/and ankle corresponding to the location of pain	For moderate pain, each treatment lasted 10 h, for severe pain, each treatment lasted 12 h, once a day for 3 weeks.
		Su et al. (40)	According to WAA theory, select the treatment area on the wrist or/and ankle corresponding to the location of pain	Each treatment lasted 9–12 h and was carried out once a day for 10 days.
		Wang et al. (42)	According to WAA theory, select the treatment area on the wrist or/and ankle corresponding to the location of pain	For moderate pain, each treatment lasted 10 h, for severe pain, the session and duration were unknown.
Ear acupoints	APA	Huang et al. (36)	Three main fixed ear acupoints: TF4, AH6a, AT4, and 6 optional points AH9, AH11, AH13, AH5, AH4, AH3, which could be selected based on the sites of bone metastases.	The ear seed tapes were changed twice a week, press each of their taped acupoints at least 6 times a day for at least 3 min every time, for 8 weeks.
		Wang et al. (41)	CO4, CO12, CO13, CO3, AH6a, TF4, AT4, HX2, CO7, AH8, CO17, and CO14 was fixed ear acupoints, other ear points could be selected based on the sites of bone metastases.	The ear seed tapes were changed once a day, press each of their taped acupoints at least nine times a day for at least 3 min every time, treatment duration was unknown.
Meridian acupoints	TEAS	Du et al. (34)	BL11, BL23, ST36, SP6.	Each treatment lasted 30 min and was carried out once a day for 4 weeks.
		Tai et al. (46)	LI4, PC6, SJ5, ST36, SP6, EX-B2.	Treatment for 5 days, twice a day.
	WA	Yao et al. (44)	ST36, RN4, RN6, Ashi.	Each treatment lasted 30 min and was carried out once a week for 4 weeks.
	MA	Lu et al. (38)	ST36, BL11, GB39, SP10, KI3, SI3, Ashi.	Each treatment lasted 30 min and was carried out once a day for 5 days.
		Zhao et al. (45)	BL2, KI3	Each treatment lasted 30 min, treatment was given for 5 days, then 2 days off, followed by treatment every day for 2 months.
	TTA	Yan (43)	BL23, BL24, BL25, KI3	Thumb-tack needles were changed once a day, press each acupoints at least 6 times a day for at least 1 min every time, for 15 days.
	CET	Gou et al. (35)	ST36, BL11, GB39, BL23, BL20, Ashi	The ACE treatment was performed every 1 weeks for a duration of 2 weeks.
Combination	APA & WAA	Ni et al. (39)	• APA: three main fixed ear acupoints:CO14, AH11, TF4, CO17, and sensitive points in ear according the different locations of pain, selected 5~6 ear points every time . • WAA: according to WAA theory, select the treatment area on the wrist or/and ankle corresponding to the location of pain	• APA: the ear seed tapes were changed twice a week, press each of their taped acupoints at least 3 times a day for at least 2 min every time, for 2 weeks . • WAA: each treatment lasted 1 h and was carried out once a day for 2 weeks.

*WAA, wrist-ankle acupuncture; TEAS, transcutaneous electrical acupoint stimulation; APA, auricular point acupressure; TTA, thumb-tack acupuncture; CET, catgut-embedding therapy; APA, auricular point acupressure; MA, manual acupuncture; WA, warm acupuncture*.

**Table 2:** Characters of the included studies.

**References**	**Country**	**Sample (EG/CG)**	**Age** ** (Mean ±SD, year)**	**Cancer type**	**Intervention**	**Control**	**Outcomes**	**Duration of intervention**	**Acupoints**
Du et al. (34)	China	24/24	UK	BM	TEAS plus any opioid	any opioid	ACDG	4 weeks	BL11, BL23, ST36, SP6.
Gou et al. (35)	China	40/40	• EG: 61.62 ± 16.11 • CG: 61.45 ± 16.28	BM	CET plus oral opioid	oral opioid	ABCH	2 weeks	ST36, BL11, GB39, BL23, BL20, Ashi
Huang et al. (36)	China	28/25	• EG: 71.7 ± 15.5 • CG: 72.4 ± 14.8	BM	APA, zoledronic acid	zoledronic acid	ACH	8 weeks	TF4, AH6a, AT4, and 6 optional points AH9, AH11, AH13, AH5, AH4, AH3, other ear points could be selected based on the sites of bone metastases.
Liu et al. (37)	China	43/40	• EG: 63.92 ± 8.47 • CG: 64.08 ± 8.52	BM	WAA plus oral opioid	oral opioid	ABEFH	≥3 weeks	According to WAA theory, select the treatment area on the wrist or/and ankle corresponding to the location of pain
Lu et al. (38)	China	30/30	• EG: 62.50 ± 0.06 • CG: 62.90 ± 9.20	BM	MA plus oral opioid	oral opioid	ABGH	5 days	ST36, BL11, GB39, SP10, KI3, SI3, Ashi.
Ni et al. (39)	China	40/40	• EG: 56.28 ± 7.10 • CG: 53.88 ± 6.23	BM	WAA, APA plus oral opioid	oral opioid	ABCHEF	2 weeks	• APA: three main fixed ear acupoints:CO14, AH11, TF4, CO17, and sensitive points in ear according the different locations of pain, selected 5–6 ear points every time. • WAA: according to WAA theory, select the treatment area on the wrist or/and ankle corresponding to the location of pain
Su et al. (40)	China	40/40	• All: 62~80 • 76.63 ± 2.51	BM	WAA	oral opioid	A	10 days	According to WAA theory, select the treatment area on the wrist or/and ankle corresponding to the location of pain
Wang et al. (42)	China	37/37	• EG: 65.25 ± 2.25 • CG: 65.27 ± 2.27	BM	WAA plus oral opioid	oral opioid	AB	UK	According to WAA theory, select the treatment area on the wrist or/and ankle corresponding to the location of pain
Yan (43)	China	60/60	• EG: 59.27 ± 15.87 • CG: 60.44 ± 15.62	BM	TTA plus oral opioid	oral opioid	AB	15 days	BL23, BL24, BL25, KI3
Yao et al. (44)	China	72/72	• EG: 67.98 ± 2.58 • CG: 67.77 ± 3.11	BM	WA plus nerve block	nerve block	ACH	4 weeks	ST36, RN4, RN6, Ashi.
Zhao et al. (45)	China	30/30	• EG: 39.27 ± 10.56 • CG: 40.00 ± 10.12	BM	MA plus zoledronic	zoledronic	ABH	8 weeks	BL2, KI3
Tai et al. (46)	China	62/65	UK	BM	TEAS	SA plus oral analgesic	A	5 days	LI4, PC6, SJ5, ST36, SP6, EX-B2.
Wang et al. (41)	China	30/30	• All: 32–80 • 62.87 ± 10.96	BM	APA plus oral analgesic	oral analgesic	ABH	UK	CO4, CO12, CO13, CO3, AH6a, TF4, AT4, HX2, CO7, AH8, CO17, and CO14 was fixed ear acupoints, other ear points could be selected based on the sites of bone metastases.

*UK, unknown; BM, bone metastases; WAA, wrist-ankle acupuncture; TTA, thumb-tack acupuncture; CET, catgut-embedding therapy; APA, auricular point acupressure; TEAS, transcutaneous electrical acupoint stimulation; MA, manual acupuncture; WA, warm acupuncture; SA, sham acupuncture; A, pain intensity; B, pain relief rate; C, adverse events; D, frequency of breakthrough pain; E, analgesic onset time; F, analgesia duration; G, Consumption of analgesics; H, quality of life*.

*: no restriction was set for opioid type, which could be an oral opioid, fentanyl, or parenteral morphine. At the end of the study, doses were converted into morphine milligram equivalents (MME)*.

### Risk of Bias

**Figure 2:**
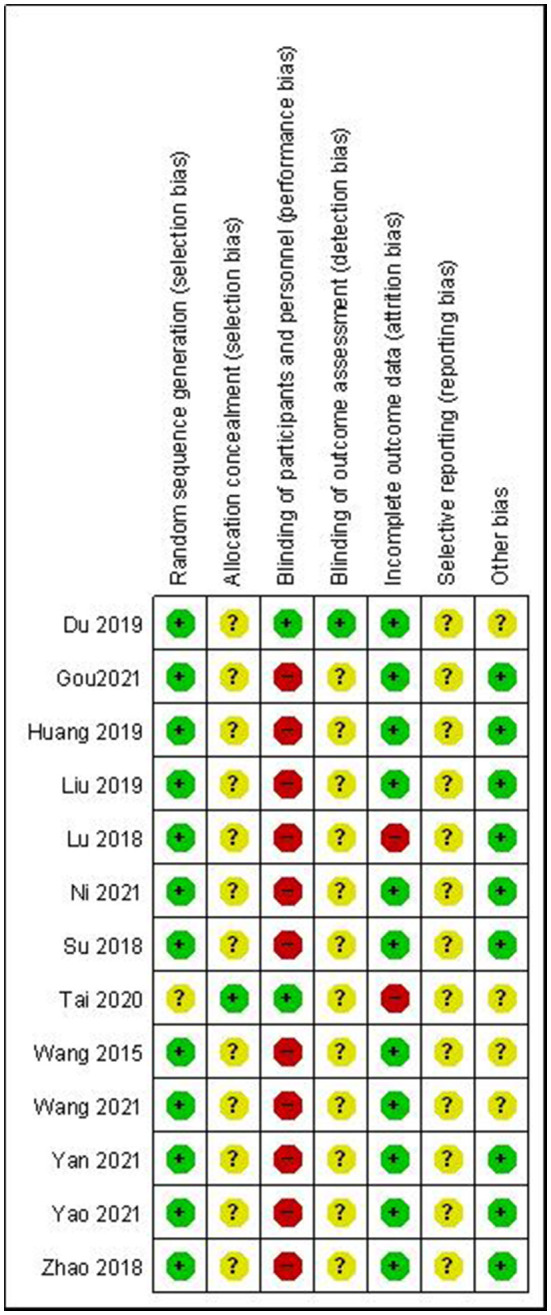
Risk of bias assessment using the Cochrane tool.

Regarding selection bias, all studies mentioned the randomization method using a random number table with a low risk of bias. Only two studies (44, 46) provided information about allocation concealment *via* the use of sealed envelopes and were low risk of allocation concealment. However, allocation concealment was unclear in the remaining studies. Acupuncture research is difficult to implement a blinding design in clinical practice owing to the special nature of acupuncture. Only two studies (34, 46) reported the blinding of patients to be rated at low risk of performance bias. Additional studies did not mention blinding design or sham acupuncture treatment and should be considered the open-label study to be rated at high risk. One study (34) mentioned the blinding of assessors and was rated at low risk of detection bias; additional studies were unclear about this domain. We assessed two studies (38, 46) as having a high risk of attrition bias for dropout rates, and attrition bias was low in the other 11 studies. Only one (46) study was registered in the Chinese Clinical Trial Registry; we obtained the evidence that there was no reporting bias from this study scheme and rated it at low risk. Additional studies were unclear about this domain. Four included studies (34, 41, 42, 46) had other biases due to the following reasons: two studies did not describe clearly the age range of the patients (34, 46) and another two studies (41, 42) did not provide the information about the treatment duration. A summary of the risk of bias in each of the included trials is presented in Figure 2.

### Pain Intensity

#### Acupuncture Plus Control Treatment vs. Control Treatment

**Figure 3:**
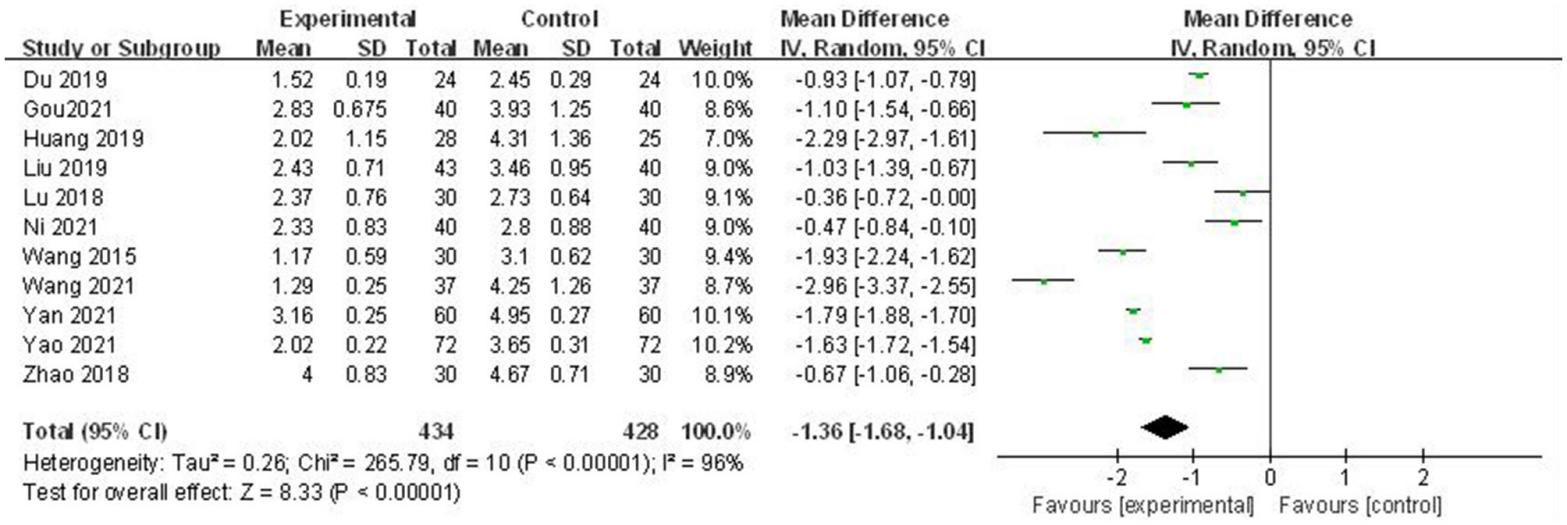
Forest plots of acupuncture plus control treatment vs. control treatment: pain intensity.

Eleven studies (34–39, 41–45) compared acupuncture plus control treatment with control treatment, and the pooled results from above studies showed a marked beneficial effect of acupuncture; however, considerable heterogeneity existed (MD = −1.34, 95% CI −1.74 to −0.94; Q < 0.1; *I*^2^ = 98%, *P* < 0.01) (see Figure 3). Given the considerable heterogeneity, we attempted to perform subgroup analyses among 11 co-intervention studies based on three perspectives (acupoints type, program length, and control group treatment) (see Table 3). The result showed that the subgroup based on acupoint type partly explained the heterogeneity, and the heterogeneity in the other two perspectives (program length and control group treatment) remained high.

**Table 3:** Results of subgroup analyses of pain score.

	***N***	**Sample size**		**MD**	**95% CI**		***Q* value**	***I* ^2^**	***P*-value**
		**EG**	**CG**		**L**	**U**			
**Acupoints type**									
Ear points	2	58	55	−1.99	−2.27	−1.71	0.35	0%	<0.01
WAA points	2	80	77	−1.99	−3.88	−0.10	<0.1	98%	0.04
Meridian points	6	256	256	−1.11	−1.49	−0.73	<0.1	97%	<0.01
Ear points plus WAA points	1	40	40	−0.47	−0.84	−0.10	–	–	0.01
**Program length**									
*n* ≤ 2 weeks	4	170	170	−0.94	−1.78	−0.09	<0.1	97%	0.03
4 weeks ≥*n* > 2 weeks	3	139	136	−1.21	−1.75	−0.66	<0.1	97%	<0.01
8 weeks	2	58	55	−1.45	−3.04	0.13	<0.1	94%	0.07
**Control group**									
Zoledronic acid	2	58	55	−1.45	−3.04	0.13	<0.1	94%	0.07
Analgesic drugs	8	304	301	−1.32	−1.80	−0.85	<0.1	97%	<0.01
Nerve block	1	72	72	−1.63	−1.72	−1.54			<0.01

#### Acupuncture Alone vs. Control Treatment

**Figure 4:**
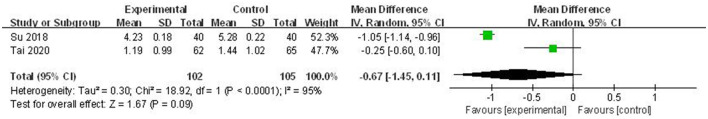
Forest plots of acupuncture alone vs. control treatment: pain intensity.

There were only two RCTs (40, 46) that investigated the effectiveness of acupuncture alone; changes in pain intensity were not significantly different between experimental groups and control groups and with strong heterogeneity (MD = −0.67, 95% CI −1.45 to 0.10; Q < 0.1; *I*^2^ = 95%, *P* = 0.09) (see Figure 4).

### Pain Relief Rate

**Figure 5:**
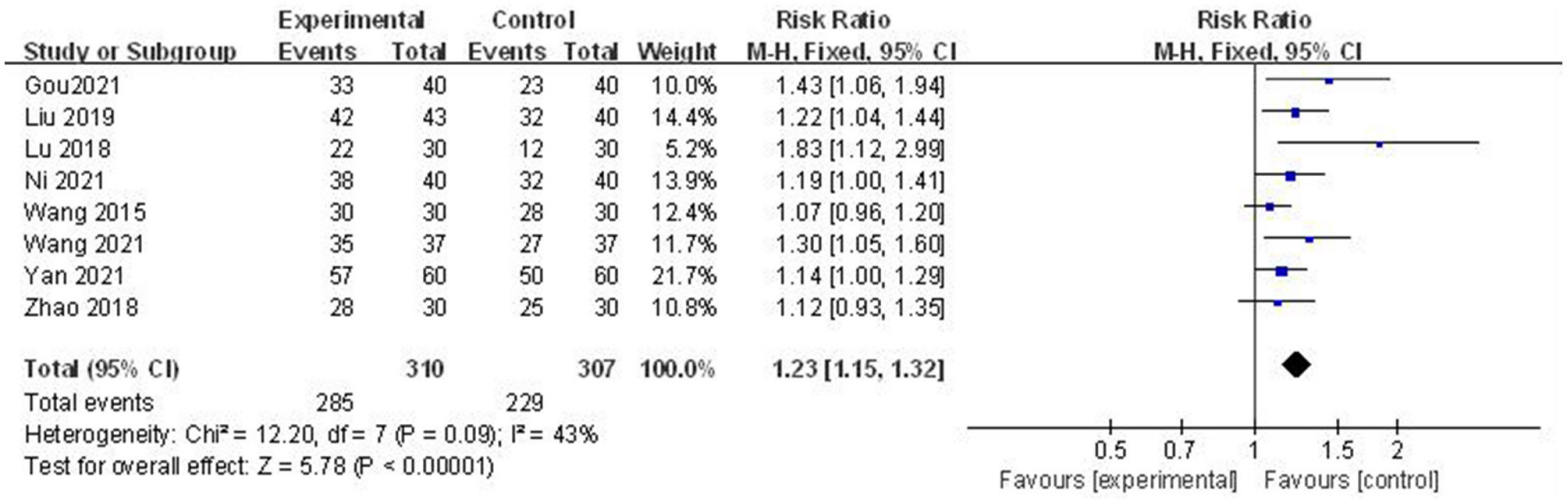
Forest plots of acupuncture plus standard treatment vs. standard treatment: pain relief rate.

Eight studies (35, 37–39, 41–43, 45) provide information about the pain relief rate after the intervention. Each study compared the efficacy of acupuncture plus control treatment vs. control treatment. The week heterogeneity (*I*^2^ = 43%, Q = 0.09) was observed among included studies. Hence, a fixed-effect meta-analysis was used to pool the data from different trials. The meta-analysis showed a statistical difference in pain relief rate between the two groups [RR: 1.23 (1.15, 1.32)]. The date are shown in Figure 5.

### Frequency of Breakthrough Pain

Only one study (34) by Du examined the frequency of breakthrough pain as an indicator for therapeutic effect evaluation. Subjects in the experimental group received treatment with TENS plus opioids, and TENS treatment was conducted once a day, whereas the control group received opioid treatments alone. Both groups received treatment for 4 weeks. The result showed that TENS plus oral opioids can significantly reduce the frequency of breakthrough pain in the experimental group compared with opioids alone (MD −0.88, 95%CI −1.07 to −0.69; *P*-value of *Z*-test < 0.01).

### Analgesic Onset Time

**Figure 6:**
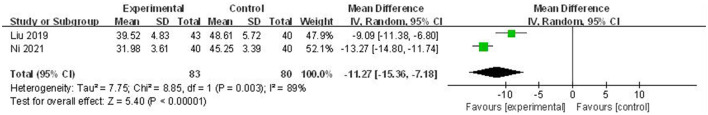
Forest plots of acupuncture plus standard treatment vs. standard treatment: analgesic onset time.

Two studies (37, 39) provide information on analgesic onset time to evaluate the effectiveness of acupuncture in pain relief. There was significant heterogeneity between the two trials (*I*^2^ = 89%, *P* = 0.10), so a random-effects model was used. The pooled data revealed that the experimental group could effectively shorten the analgesic onset time compared to the control group [MD: −11.27 (−15.36, −7.18)]. The date were shown in Figure 6.

### Analgesia Duration

**Figure 7:**
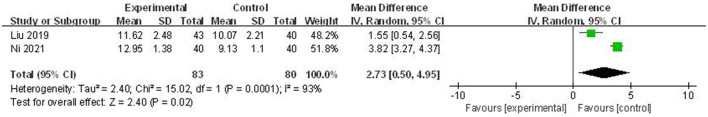
Forest plots of acupuncture plus standard treatment vs. standard treatment: analgesic duration.

The reporting on the analgesia duration was provided in two studies (37, 39). We performed a descriptive analysis since the heterogeneity (*I*^2^ = 93%, *P* < 0.1) was signed between the two studies, and the study by Liu et al. (37) showed that the time to analgesia duration was significantly longer in the experimental group than in the control group (MD 1.55, 95% CI 0.54–2.56; *P*-value of *Z*-test = 0.03). In the study by Ni et al. (39), there was the same trend for the experimental group to show longer analgesia duration than the control group (MD 3.82, 95% CI 3.27–4.37; *P*-value of *Z* test < 0.00001). The date are shown in Figure 7.

### Quality of Life

Eight studies (35–39, 41, 44, 45) evaluated the quality of life of patients across four different scales. We only pooled the data of the same scale due to the large differences in the evaluation methods among the scales. EORTC QLQ-C30 was chosen by three studies (37, 39, 44) comprising 30 items grouped into 15 scales (47). However, in these three studies, researchers selected different items among 30 items to assess the quality of life in cancer patients. The study by Liu et al. (37) used four items: physical functioning, pain, insomnia, and global health status. The study by Ni et al. (39) selected five items: physical functioning, cognitive, pain, emotional, and global health status. The study by Yao et al. (44) also chose five items: physical functioning, cognitive, emotional, social functioning, and global health status. Hence, we performed meta-analyses based on the above metrics' data. These meta-analyses show that acupuncture treatment effectively affects global health status, physical functioning, pain, emotional, insomnia, and social functioning. KPS scale was used in the other three studies (35, 38, 41), and the pooled data showed that patients in the experimental group had a higher score than controls (MD = 9.85, 95% CI 3.18–16.52; Q = 0.03; *I*^2^ = 72%, *P* = 0.004). PROSQOL scale was used in the study by Huang et al. (36), which is a multidimensional scale and includes nine domains: pain, strength, appetite, urination, the degree of fatigue, constipation, the relationship of marriage/family, emotional, and general feeling. The clinical research showed that auricular point acupressure plus zoledronic acid was effective in pain, appetite, emotions, and general feeling but may be ineffective in other domains. The ECOG scale was used in the study by Zhao et al. (45), and the score is inversely associated with quality of life. The result shows that manual acupuncture plus zoledronic acid resulted in lower scores than zoledronic acid treatment alone. Please see Table 4 for further detail.

**Table 4:** Details of scales of quality of life.

**Scale**	**Domain**	***N***	**Sample size**		**MD**	**95% CI**		***Q*-value**	***I* ^2^**	***P*-value**
			**EG**	**CG**		**L**	**U**			
EORTC QLQ-C30	Global health status	3	155	152	9.61	8.44	10.78	0.66	0%	<0.01
	Physical functioning	3	155	152	6.79	1.27	12.31	<0.1	92%	0.02
	Pain	2	83	80	−12.05	−23.84	−0.26	<0.1	99%	0.05
	emotional	2	112	112	6.28	4.30	8.27	0.13	56%	<0.01
	cognitive	2	112	112	5.44	−5.82	16.70	<0.1	97%	0.34
	insomnia	1	43	40	−10.61	−13.07	−8.15	–	–	<0.01
	Social functioning	1	72	72	6.63	4.89	8.37	–	–	<0.01
PROSQOL	Pain	1	28	25	9.05	4.19	13.91	–	–	0.0003
	Strength	1	28	25	4.65	−1.13	10.43	–	–	0.11
	Appetite	1	28	25	7.10	−0.13	14.33	–	–	0.05
	Urination	1	28	25	1.17	−5.59	7.93	–	–	0.73
	The degree of fatigue	1	28	25	3.96	−3.42	11.34	–	–	0.29
	Constipation	1	28	25	3.0	−3.57	9.57	–	–	0.37
	Relationship of marriage/family	1	28	25	0.78	−5.70	7.26	–	–	0.81
	Emotional	1	28	25	6.68	−1.53	14.89	–	–	0.11
	General feeling	1	28	25	10.02	3.23	16.89	–	–	0.004
KPS	–	3	100	100	9.85	3.18	16.52	0.03	72%	0.004
ECOG	–	1	30	30	−0.42	−0.68	−0.16	–	–	0.002

### Safety

Four (34, 35, 39, 44) of the included studies reported the occurrence of adverse events. The adverse events reported by these studies were largely related to opioids' side effects, including nausea or vomiting, constipation, dizziness, itching, delirium, drowsiness, and abdominal distention, and there is no report about the adverse effects associated with acupuncture treatment. In the study by Du (34), four kinds of mild adverse events were reported, nausea and constipation occurred less frequently in the experimental group than in the control group, while the incidence of dizziness and itching was similar in the experimental group and control group. Ni et al. (39) also reported some mild adverse events, including nausea, dizziness, constipation, and itching. The incidence of adverse events was lower in the experimental group (7.5%) than in the control group (17.5%). The study by Yao et al. (44) reported four kinds of adverse events, including constipation, nausea, delirium, and drowsiness. The incidence of adverse events in the experimental group was 5.6%, significantly lower (*P* < 0.05) compared with the control group (19.4%). The study by Gou et al. (35) reported four adverse events, including nausea or vomiting, constipation, drowsiness, and abdominal distention. The incidence of adverse events in the experimental group was 2.5%, significantly lower (*P* < 0.05) compared with the control group (22.50%). The details of adverse events are shown in Table 5.

**Table 5:** Details of adverse events.

**References**	**Group**	**Nausea or vomiting**	**Constipation**	**Dizziness**	**Itching**	**Delirium**	**Drowsiness**	**Abdominal**
								**distention**
Du et al. (34)	Experimental group	3	13	2	0	0	0	0
	Control group	9	20	2	1	0	0	0
Ni et al. (39)	Experimental group	1	0	1	1	0	0	0
	Control group	2	2	2	1	0	0	0
Yao et al. (44)	Experimental group	1	2	0	0	1	0	0
	Control group	4	4	0	0	3	3	0
Gou et al. (44)	Experimental group	0	0	1	0	0	0	0
	Control group	2	3	1	0	0	0	1

### GRADE Evaluation of Certainty of Outcomes

The GRADE system was used to assess the quality of evidence for the outcomes in this review. In the present study, every outcome was qualified as “very low.” From the perspective of methodological quality criteria, most included studies failed to perform effective blinding or allocation concealment, which may result in a risk of bias. Inconsistency might exist because different methods measured the outcomes, and several acupuncture techniques were used across included studies. Some meta-analyses included a low number of studies which added the imprecision of evidence. Considerable heterogeneity existing in most meta-analyses was associated with the increase in inconsistency. The details are shown in Table 6.

**Table 6:** GRADE evidence profile of outcomes.

**Quality assessment**							**No of patients**		**Effect**		**Quality**	**Importance**
**No of studies**	**Design**	**Risk of bias**	**Inconsistency**	**Indirectness**	**Imprecision**	**Other considerations**	**Pain score**	**Control**	**Relative (95% CI)**	**Absolute**		
**Acupuncture plus control treatment vs. control treatment (measured with: VAS/NRS/BPI; Better indicated by lower values)**												
11	Randomized trials	Very serious^a^	Very serious^b^	No serious indirectness	Serious^c^	• Reporting bias^d^ • Strong association^e^	434	428	–	MD 1.34 lower (1.74–0.94 lower)	⊕OOO Very low	Critical
**Acupuncture alone vs. control treatment (measured with: VAS; Better indicated by lower values)**												
2	Randomized trials	Very serious^f^	Very serious^b^	No serious indirectness	Serious^c^	None^g^	102	105	–	MD 0.67 lower (1.45 lower to 0.11 higher)	⊕OOO Very low	Critical
**Pain relief rate (assessed with: Based on the pain score)**												
8	Randomized trials	Very serious^a^	Serious^h^	No serious indirectness	Serious^c^	None^g^	285/310 (91.9%)	229/307 (74.6%)	OR 4.4 (2.64–7.33)	182 more per 1,000 (from 140 more to 210 more)	⊕OOO Very low	Important
								80%		146 more per 1,000 (from 113 more to 167 more)		
**Analgesic oneset time (measured with: Analgesic oneset time; Better indicated by lower values)**												
2	Randomized trials	Very serious^i^	Very serious^b^	No serious indirectness	Serious^c^	None^g^	83	80	–	MD 11.27 lower (15.36–7.18 lower)	⊕OOO Very low	Important
**Analgesia duration (measured with: Analgesia duration; Better indicated by lower values)**												
2	Randomized trials	Very serious^i^	Very serious^b^	No serious indirectness	Serious^c^	None^g^	83	80	–	MD 3.3 higher (2.82–3.79 higher)	⊕OOO Very low	Important
**Global health status (measured with: EORTC QLQ-C30; Better indicated by lower values)**												
3	Randomized trials	Very serious^a^	No serious inconsistency	No serious indirectness	Serious^c^	None^g^	155	152	–	MD 9.61 higher (8.44–10.78 higher)	⊕OOO Very low	Not important
**Physical functioning (measured with: EORTC QLQ-C30; Better indicated by lower values)**												
3	Randomized trials	Very serious^a^	Very serious^b^	No serious indirectness	Serious^c^	None^g^	155	152	–	MD 6.79 higher (1.27–12.31 higher)	⊕OOO Very low	Not important
**Pain (measured with: EORTC QLQ-C30; Better indicated by lower values)**												
2	Randomized trials	Very serious^a^	Very serious^b^	No serious indirectness	Serious^c^	None^d^	83	80	–	MD 12.05 lower (23.84–0.26 lower)	⊕OOO Very low	Not important
**Emotional (measured with: EORTC QLQ-C30; Better indicated by lower values)**												
2	Randomized trials	Very serious^a^	No serious inconsistency	No serious indirectness	Serious^c^	None^d^	112	112	–	MD 6.28 higher (4.3–8.27 higher)	⊕OOO Very low	Not important
**Cognitive (measured with: EORTC QLQ-C30; Better indicated by lower values)**												
2	Randomized trials	Very serious^a^	Very serious^b^	No serious indirectness	Serious^c^	None^d^	112	112	–	MD 5.44 higher (5.82 lower to 16.7 higher)	⊕OOO Very low	Not important
**KPS (measured with: KPS; Better indicated by lower values)**												
3	Randomized trials	Very serious^a^	Serious^b^	No serious indirectness	Serious^c^	None^d^	100	100	–	MD 9.85 higher (3.18–16.52 higher)	⊕OOO Very low	Not important

a *These studies were affected by several factors, such as performance bias, detection bias, and reporting bias*.

b *There was high heterogeneity across studies*.

c *The sample was small*.

d *Exist publication bias*.

e *The number of total patients was over 800*.

f *The study by Su had selection bias, performance bias, detection bias, and reporting bias; the study by Tai had detection bias, attrition bias, and other bias*.

g *The number of included studies was small*.

h *Apply different acupuncture techniques across studies*.

i *Two studies did not mention allocation concealment, blinding, and have reporting bias*.

## Discussion

### Summary of the Main Results

As far as we know, this is the first systematic review and meta-analysis to evaluate acupuncture's effectiveness in treating CIBP. Clinically, CIBP patients not only experienced background chronic pain but also suffer from acute pain termed breakthrough pain (7); therefore, timely and consistent relief from pain intensity is necessary to manage CIBP effectively. This systematic review intends to make a comprehensive evaluation of the effectiveness of acupuncture on CIBP, not only assessing the pain score and pain relief rate, but also the frequency of breakthrough pain, analgesic onset time, and analgesic duration. Pain score was the primary outcome in the present review; our findings showed that acupuncture plus control treatment improved effectiveness in reducing pain intensity in patients with CIBP, whereas acupuncture treatment alone failed to do that. In addition, it significantly increased the pain relief rate, reduced the frequency of breakthrough pain, shortened analgesic onset time, and extended the analgesic duration when the control groups added acupuncture therapy. Therefore, the outcome of this systematic review supported the effectiveness of acupuncture as adjunctive therapy for pain management in CIBP. We did not obtain enough evidence about acupuncture alone treatment to prove its effectiveness, and the inclusion of only two studies is one of the possible explanations for the observed non-significant treatment effects. It was worth noting that the overall quality of evidence was rated as very low. Consistent with the results of previous meta-analyses (27, 48), there is a synergistic effect between acupuncture and conventional treatment in mitigating cancer pain. Cancer pain is of complex mechanisms that are not well-controlled by conventional opioid analgesics (7). It should be pointed out that acupuncture should be applied as a complementary medical therapy rather than an alternative therapy for cancer pain management (49). Our findings demonstrated that acupuncture might have advantages in reducing the frequency of breakthrough pain, shortening analgesic onset time, and extending the analgesic duration. However, it should be noted that corresponding meta-analyses included a small number of RCTs (*n* = 2) and the effect of acupuncture as adjunctive therapy may have been overestimated, and the overall quality of evidence was rated as very low. We believe these metrics are important in evaluating the effectiveness of acupuncture in CIBP and should obtain more attention in future RCTs. There was considerable heterogeneity among included studies, and subgroup analysis based on acupoints type partly explained the sources of heterogeneity. Notably, seven kinds of acupuncture treatments have been used across 13 included studies, complicating this system review's analyses. The change in the acupuncture technique brings a corresponding change in the treatment frequency, duration of treatment, needle retention time, number of sessions, and needling depth, all of which might have been potential biasing factors. Given that studies concerning acupuncture's effectiveness on CIBP are less common, we had to use less restricted inclusion criteria to retrieve relevant RCT studies. In the future, more RCTs focusing on this matter should be carried out to confirm the effectiveness of acupuncture on CIBP.

In recent years, many researchers or institutions have been appealing to pay more attention to the quality of life in cancer patients than just killing cancer itself (50). Previous studies found that acupuncture can improve the quality of life of cancer patients (51, 52). Cancer pain is the major factor affecting cancer patients' quality of life, and it has been found that the stronger the intensity and the higher the frequency of pain, the lower the quality of life (53). Hence, we believe that the improvement in quality of life reflects the effectiveness of cancer pain management. In this system review, eight studies evaluated patients' quality of life and reported similar findings that patients in the experimental group had a better quality of life. The quality of life provides indirect evidence of the effectiveness of acupuncture treatments on CIBP. However, eight studies used four scales, and there was a large variation in the evaluation methods across the scales; pooling data from different scales was not always appropriate. In the end, we performed a meta-analysis based on data from three studies using the EORTC QLQ-C30 scale and the other three using the KPS scale. The results showed that acupuncture could improve CIBP patients' global health status, physical functioning, pain, emotional, insomnia, social functioning, and KPS scores. Thus, future studies should use a uniform and valid scale to assess cancer patients' quality of life to facilitate comparison and data pooling across studies. Opioids are an irreplaceable analgesic in managing cancer pain, whereas long-term opioid use leads to unwanted side effects, such as constipation, respiratory depression, addiction, and tolerance, which diminish the quality of life (11). How to reduce the side effects of opioids and improve therapeutic efficacy remains a major challenge. It should be noted that adverse events reported in this review were largely related to opioids' side effects rather than acupuncture treatment, and the incidence of side effects of opioids was lower than that in the control group. This evidence showed that acupuncture is a safe and well-tolerated treatment.

### The Application of Acupoints

According to the theory of TCM, the efficacy of acupuncture is affected by the selection of acupoints, which is changeable according to the condition of the patients, the experience of the acupuncturists, and the type of acupuncture (54). The studies included in the present meta-analysis used seven acupuncture techniques. WAA was used for four studies (37, 39–41), a modern acupuncture therapy performed through the subcutaneous insertion of needles at points on the wrist and ankle regions (55). Wrist-ankle acupuncture features simple operation, no pain, and easier acceptance by patients (27, 56). A meta-analysis in 2021, including 13 RCTs, revealed that WAA has a certain effect on cancer pain (27). Subgroup analysis showed that the effect of WAA plus analgesic drug on CIBP relief was statistically significant in relieving cancer pain compared with analgesic drug alone (*P*=0.04, CI−3.88 to−0.10). Auricular point acupressure is the method of stimulating specific points on the ear by applying pressure to them (21). This method was used in three studies (36, 39, 41). The acupuncture prescriptions of the three studies were different, and researchers select ear points based on the sites of bone metastases; however, these studies chose the same ear point, ear Shenmen (TF4), which is a key point for pain relief (57). Five other acupuncture therapies were used in the remaining seven studies (34, 35, 38, 43–46), including thumb-tack acupuncture, manual acupuncture, warm acupuncture, TEAS, and catgut-embedding therapy. Although stimulating acupoints were different among the above acupuncture techniques, the range of selecting acupoints was the same, based on meridian points. Since meridians and collaterals criss-cross to network the whole body, the stimuli of acupuncture on acupoints can not only play a positive role in local treatment but also modulate systemic physiology. These studies all used a predetermined set of acupoints; the number of acupoints in each study ranged from two to seven. A total of 19 acupoints were used from seven studies and recorded 33 times (see Table 7). The top five most used acupoints were ST36, BL23, KI3, BL11, and Ashi. Traditional Chinese Medicine (TCM) believes that cancer is a disease that combines deficiency and excess. In addition, the kidney is associated with bone in constituents; bone health is affected by the kidney's essence. The deficiency of kidney essence results in bone losing its nourishment and cancer toxin invading in bone, which is taken as the major pathogeneses of bone metastases based on Chinese medicine theory. Five studies selected ST36, a kind of energy-associated point belonging to the Stomach Meridian of Foot Yangming, specifically applied to assisting the vital qi and building a good physique. BL23, KI3, and BL11 appeared three times, the back-shu acupoint of kidney organ, yuan-source of kidney meridian, and one of eight influential acupoints, respectively. Acupuncture at these three points can nourish the kidneys and strengthens the bones, strengthening the vital to dispel the pathogen, and arrived at alleviating CIBP. The abovementioned four points mainly reflect the systemic therapeutic effect of acupuncture, while the Ashi reflects the role of acupuncture in local treatment. Ashi points refer to those which can produce a painful or comfortable sensation when pressed appropriately (58), which are mainly used for pain relief. However, based on our study, only three studies used the Ashi point. It is worth noting that bone metastasis will cause not only intractable bone pain but also cause great bone destruction, leading to pathological fractures (59), which pose a great challenge to acupuncture at Ashi. As acupuncture treatment is an automatic stimulation, the safety of acupuncture in CIBP needs to be a concern, that is, whether acupuncture at Ashi will increase local bone tissue damage and promote disease progression. However, the three studies included in this study did not report any event about disease progression. The number of studies we have included is relatively small. Our research failed to provide sufficient evidence for acupoint selection rules for acupuncture treatment of CIBP, but it can still provide a reference for scholars. To some extent, the RCTs in our study pay more attention to the systemic therapeutic effect of acupuncture. Whether Ashi acupoints should be used for the treatment of CIBP and the most suitable acupuncture prescription for CIBP treatment would be interesting areas for further research.

**Table 7:** Details of meridian acupoints.

**Cupoint**	**Counts**	**Acupoint**	**Counts**	**Acupoint**	**Counts**	**Acupoint**	**Counts**
ST36	5	GB39	2	BL24	1	BL2	1
BL23	3	SP6	2	BL25	1	LI4	1
BL11	3	BL20	1	RN4	1	SJ5	1
KI3	3	SP10	1	RN6	1	PC6	1
Ashi	3	SI3	1	EX-B2	1		

### Limitations of the Current Study

This study has several limitations. First of all, there is a certain inclusion bias in this study. On the one hand, the literature search was limited to articles published in Chinese or English, excluding studies published in other languages such as Japanese, Korean, and German; on the other hand, in most cases, positive trials are published rather than negative ones; however, negative findings had important implications for our research. We had not retrieved studies with negative results. More avenues should be tried in the future, looking for negative reports. Second, CIBP is the most common type of cancer pain. The subjects included in the current review were all cancer patients with bone metastases, which always indicates that patients presented an advanced tumor stage. Primary cancer may also be a source of cancer pain at this stage. Cancer pain other than CIBP may have significantly influenced study outcomes. However, some studies (34, 36, 38, 40–43, 45, 46) included in this review do not explicitly state that pain in cancer patients only includes CIBP. Future clinical studies should strictly design inclusion and exclusion criteria to reduce the influence of irrelevant factors on the results. Third, the studies included in this review were not of high methodology quality. According to the seven risks of bias domains of the Cochrane collaboration tool, no studies were assessed as having a low risk of bias across all domains. Only two studies described the method of allocation concealment; 10 studies lacked blinding design; two reported dropout events; only one was registered in the Chinese Clinical Trial Registry. We obtained evidence that there was no reporting bias in this study from the research scheme. However, we were not sure about other studies. The above bias interfered with the validity and reliability of the outcome to a varying degree and resulted in a very low quality of evidence. We strongly suggest that researchers should provide a reasonable design of randomization, allocation concealment, and blinding for future RCTs. Finally, all 13 studies included a relatively small sample size (*n* = 48–144), which was likely to reduce the outcomes' precision and produce misleading results. Based on the analysis above, future RCTs should include greater numbers of participants, be registered on relevant online platforms, disclose necessary information to improve the transparency and authenticity of clinical trials, and facilitate the provision of reference information for scientific researchers.

## Conclusion

Acupuncture has a certain effect as a complementary therapy on pain management of CIBP, which not only mitigates the pain intensity but also improves the quality of life, and reduces the incidence of opioid side effects. Due to the poor quality of methodology and strong heterogeneity in the included studies, the results of this meta-analysis should be interpreted with caution. Regarding acupoint selection, the systemic therapeutic effect of acupuncture obtains more attention, and ear points, wrist-ankle acupoints, ST36, BL23, BL11, and KI3 are essential for the acupoint selection. In addition, whether Ashi Point is suitable for treating CIBP still needs further exploration. In future, a large sample size and rigorously designed RCTs are needed to provide sufficient evidence to identify the effectiveness and safety of acupuncture as a treatment for CIBP. Meanwhile, researchers should gradually explore the law of acupuncture treatment for CIBP, including the choice of acupuncture techniques, acupoints, needle retention time, and sessions, in hopes of promoting the application of acupuncture in the pain management of CIBP.

## Data Availability Statement

The original contributions presented in this study are included in the article/Supplementary Material, further inquiries can be directed to the corresponding authors.

## Author Contributions

MaL and MiL conceived and designed the research. HZ contributed to the design of a research strategy. ZY, ZM, and MaL contributed to the literature search, data extraction, and assessment of risks of bias. CY and HZ performed the statistical analysis, interpreted the data, and discussed the format and content of the article and contributed to the review and editing of the final manuscript. ZY and ZM wrote the first draft of the manuscript. MiL and CY revised the manuscript for intellectual content. All authors revised the manuscript for important intellectual content and read and approved the final manuscript.

## Funding

This study was supported by the National Natural Science Foundation of China (Grant No. 82074559), Key Scientific Research Foundation of Education Bureau of Hunan Province, China (Grant No. 21A0235), Scientific and Technological Personnel lift project in Hunan Province (Grant No. 2019TJ-Q04), Furong Scholars Award Program in Hunan Province (Xiang Jiao Tong [2020] 58.), and Training Program for Excellent Young Innovators of Changsha (Grant No. kq1905036).

## Conflict of Interest

The authors declare that the research was conducted in the absence of any commercial or financial relationships that could be construed as a potential conflict of interest.

## Publisher's Note

All claims expressed in this article are solely those of the authors and do not necessarily represent those of their affiliated organizations, or those of the publisher, the editors and the reviewers. Any product that may be evaluated in this article, or claim that may be made by its manufacturer, is not guaranteed or endorsed by the publisher.
