# Supplementary material for: Acupuncture as a Complementary Therapy for Cancer-Induced Bone Pain: A Systematic Review and Meta-Analysis
Source: Front Pain Res (Lausanne). 2022 Aug 1;3:925013. doi: 10.3389/fpain.2022.925013 (PMC9377524; doi:10.3389/fpain.2022.925013)
Supplement: Supplementary file 1 [file Data_Sheet_1.pdf]

Supplementary Material

Section and Topic	Item #	Checklist item	Location where item is reported
**TITLE**			
Title	1	Acupuncture as a Complementary Therapy for Cancer-Induced Bone Pain: a Systematic Review and Meta-analysis	1
**ABSTRACT**			
Abstract	2	Background: Cancer-induced bone pain (CIBP) is a special type of cancer pain and lacks safe and effective treatments. Acupuncture is a potentially valuable treatment for CIBP, studies evaluating the effect of acupuncture on CIBP have increased significantly, but the safety and efficacy of acupuncture to control CIBP remains controversial.<br><br>Objective To provide the first meta-analysis to evaluate the safety and efficacy of acupuncture in CIBP management.<br><br>Data sources CNKI, CBM, Wanfang, VIP Database, PubMed, Embase, and Cochrane Library were searched from their inception until June 1, 2022.<br><br>Study selection RCTs with primary bone tumor patients or other types primary of cancer companied by bone metastases as the research subjects and to evaluate the efficacy of acupuncture treatment alone or combined with the control treatment were included. Meanwhile, RCTs should choose the pain score as the primary outcome and pain relief rate, frequency of breakthrough pain, analgesic onset time, analgesia duration, quality of life, and adverse events as reference outcomes.<br><br>Data collection and analysis We designed a data-extraction form that was used to extract key information from the articles. Data extraction studies evaluation was conducted independently by two reviewers, a third reviewer would resolve any disagreements. The risk of bias was assessed by the Cochrane Collaboration's tool for assessing the risk bias. Mean differences (MD), relative risk (RR) and 95% confidence intervals (CIs) were calculated. We did separate random-effects meta-analyses for pain intensity, pain relief rate, frequency of breakthrough pain, analgesic onset time, analgesia duration, quality of life. The quality of the evidence for main outcomes was evaluated by the GRADE system. The forest plots were performed using Review Manager Software (5.3 version). Subgroup analysis was used to investigate the possible sources of potential heterogeneity. Descriptive analysis was performed in case of unacceptable clinical heterogeneity.<br><br>Results Thirteen RCTs (with 1069 patients) were included, and all studies were at high risk of bias owing to lack of blinding or other bias. Eleven studies evaluated the effectiveness of acupuncture as a complementary therapy, and showed that acupuncture plus control treatment (compared with control treatment) was connected with reduced pain intensity (MD = -1.34, 95% CI -1.74 to -0.94; Q<0.1; I2 = 98%, P<0.01). Subgroup analyses based on acupoints type partly explain the potential heterogeneity. The results also showed that acupuncture plus control treatment (compared with control treatment) was connected with relieving pain intensity, increasing the pain	1~2

Section and Topic	Item #	Checklist item	Location where item is reported
		relief rate, reducing the frequency of breakthrough pain, shortening analgesic onset time, extending the analgesic duration, and improving the quality of life. We have no sufficient evidence to prove the effectiveness of acupuncture alone. Four RCTs reported only adverse events related to opioids' side effects. Evidence was qualified as "very low" because of low methodological quality, considerable heterogeneity or a low number of included studies.<br><br>Conclusion Acupuncture has a certain effect as a complementary therapy on pain management of CIBP, which not only mitigates the pain intensity, but also improves the quality of life and reduces the incidence of opioids' side effects, although the evidence level was very low. In the future, larger sample size and rigorously designed RCTs are needed to provide sufficient evidence to identify the efficacy and safety of acupuncture as a treatment for CIBP.<br><br>Funding This study was supported by grants from the National Natural Science Foundation of China (Grant Nos. 82074559), Key Scientific Research Project of Hunan Education Department (Grant Nos. 21A0235), Hunan Science and Technology Talents Project (Grant Nos. 2019TJ-Q04), Hunan Province "Lotus Scholar Award Program" (Xiang Jiao Tong [2020] 58.), Training Program for Excellent Young Innovators of Changsha (Grant Nos. kq1905036).	
**INTRODUCTION**			
Rationale	3	Describe the rationale for the review in the context of existing knowledge.<br><br>Cancer-induced bone pain (CIBP) is a common source of moderate and severe cancer pain. When cancer invades bone and surrounding tissues, cancer cells can release pain mediators and cause peripherally and centrally neuropathic changes, which contribute to a mixed-mechanism pain state. Bone metastasis is the most contributor to CIBP, which is a common symptom in advanced cancers. CIBP exists as a combination of background and breakthrough pain. Breakthrough pain is characterized by rapid onset and short duration, which limits the efficacy of standard analgesics in controlling CIBP. Despite this, oral morphine is still an important choice for patients who can receive oral medication. In addition, long-term opioid use produced unwelcome side effects, which result in a reduction in patient compliance and negatively impact on patient activities of daily living. Hence, there is an urgent need to explore an effective treatment with fewer side effects to alleviate CIBP.<br><br>Acupuncture is an ancient traditional Chinese medical practice involving stimulating specific acupoints and meridian channels to control pain and other symptoms. A variety of acupuncture techniques have been devised in clinical use, including manual acupuncture, auricular point acupressure, moxibustion, catgut-embedding therapy, and so on. The effect on pain-relieving of acupuncture has been accepted by leading organizations in the medical community, such as the American Society for Clinical Oncology and the National Comprehensive Cancer Network, and acupuncture, as a non-pharmacological intervention, has been widely used for cancer pain management. Acupuncture for cancer pain has been a research hot spot in the field of cancer research. Many systematic reviews have established the association between acupuncture and cancer pain. However, no recent systematic reviews or meta-analyses have focused on the safety and efficacy of acupuncture for CIBP. Research on acupuncture for CIBP has continued in recent years, but the findings have been inconsistent. In addition, the use of acupuncture to control CIBP remains controversial, and there is a concern that acupuncture may increase tumor growth. Therefore, this analysis aimed to evaluate the efficacy and safety of acupuncture in CIBP management based on current relevant RCTs to provide scientific references for future research and practice.	2~3

Section and Topic	Item #	Checklist item	Location where item is reported
Objectives	4	Provide an explicit statement of the objective(s) or question(s) the review addresses.<br>① To evaluate the benefits and harms of acupuncture (as a complementary treatment or alternative treatment) on pain intensity, pain relief rate, frequency of breakthrough pain, analgesic onset time, analgesia duration, and quality of life compared with conventional analgesia (oral morphine, morphine injection, fentanyl, parenteral morphine, zoledronic acid, nerve block) in people with CIBP.<br>② Investigated the experience and precautions of acupoint selection in treating CIBP with acupuncture.	3
METHODS			
Eligibility criteria	5	Specify the inclusion and exclusion criteria for the review and how studies were grouped for the syntheses.<br><br>The inclusion criteria were (1) study design: only randomized controlled trials (RCTs) were eligible, and (2) participants: patients were diagnosed with primary bone cancer or other types of primary cancer companied by bone metastases. CIBP patients were diagnosed by imaging or biopsy. There was well-defined localized pain in CIBP patients, and (3) the number of subjects in each group of one RCT should be greater than or equal to 20, and (4) intervention and control: the intervention group received at least one of the following acupuncture treatments: manual acupuncture, auricular point acupressure, moxibustion, catgut-embedding therapy, thumb-tack acupuncture, wrist-ankle acupuncture, transcutaneous electrical acupoint stimulation, warm acupuncture, electroacupuncture with or without the combination of the control treatment, regardless of acupoints, frequency, sessions. Treatments in the control group can be oral morphine, morphine injection, fentanyl, parenteral morphine, zoledronic acid, nerve block, sham acupuncture, placebo, or usual care, and (5) outcome measures: pain intensity was described as the primary outcome, which should be reported in each included study. Pain intensity can be measured by a pain measurement, such as the Visual Analog Scale for Pain (VAS Pain), the Numeric Rating Scale for Pain (NRS Pain), or the item of "the current pain item" in the Brief Pain Inventory (BPI). We determined pain relief rate, frequency of breakthrough pain, analgesic onset time, analgesia duration, quality of life, and adverse events as reference outcomes.<br><br>The exclusion criteria were (1) studies were related to qualitative studies, animal studies, case reports, or other topics not related to RCTs, and (2) the same study, which was republished, and (3) studies with incomplete original data, or data that could not be extracted and still unavailable after contacting the authors, and (4) there are obvious errors in the outcome, and the statistical methods are inappropriate, and (5) when experimental group applies a pain relief method which is different from the control group.<br><br>The meta-analysis was performed using Review Manager Software (5.3 version). The effect size of dichotomous and continuous data was presented as relative risk (RR) and mean difference (MD); both were reported with a 95% confidence interval. The heterogeneity among trials was identified by the $\chi 2$ test and quantified by the I^2 statistic. When heterogeneity test was acceptable ($P > 0.1$, $I2 \leq 50\%$), a fixed-effects model was used for meta-analysis. When the heterogeneity was significant ($P \leq 0.1$, $I2 > 50\%$), a random-effects model was used for meta-analysis. Subgroup analyses would be performed to analyze the source of heterogeneity. Descriptive analyses should be selected instead of a meta-analysis if P < 0.1 and the sources of diversity are unknown. Pooled effects were calculated, and a 2-sided P value <0.05 was considered to indicate statistical significance.	3~4

Section and Topic	Item #	Checklist item	Location where item is reported
Information sources	6	Specify all databases, registers, websites, organisations, reference lists and other sources searched or consulted to identify studies. Specify the date when each source was last searched or consulted.<br>Seven databases were searched from their inception through June 1, 2022:<br>① China National Knowledge Infrastructure (CNKI): n=152;<br>② Chinese Biomedical Literature Database (CBM): n=136;<br>③ Wanfang: n=117;<br>④ VIP: n=64;<br>⑤ PubMed: n=32;<br>⑥ Embase: n=38;<br>⑦ Cochrane Library: n=43.	3
Search strategy	7	Present the full search strategies for all databases, registers and websites, including any filters and limits used.<br>CNKI:<br>(Professional search)<br>TKA=('骨癌痛'+'癌性骨痛'+'骨转移痛'+'骨癌疼痛'+'骨转移'+'骨肉瘤'+'骨癌'+'软骨肉瘤'+'恶性骨肿瘤')*('针刺'+'针灸'+'电针'+'温针'+'揿针'+'经皮神经电刺激'+'耳穴'+'埋线'+'腕踝针'+'艾灸')<br>Wanfang:<br>(Professional search)<br>题名或关键词: ("骨癌痛" or "癌性骨痛" or "骨转移痛" or "骨癌疼痛" or "骨转移" or "骨肉瘤" or "骨癌" or "软骨肉瘤" or "恶性骨肿瘤") and 题名或关键词:("针刺" or "针灸" or "电针" or "温针" or "揿针" or "经皮神经电刺激" or "耳穴" or "埋线" or "腕踝针" or "艾灸")<br>VIP:<br>(structure search.)<br>M=(骨癌痛 OR 癌性骨痛 OR 骨转移痛 OR 骨癌疼痛 OR 骨转移 OR 骨肉瘤 OR 骨癌 OR 软骨肉瘤 OR 恶性骨肿瘤) AND M=(针刺 OR 针灸 OR 电针 OR 温针 OR 揿针 OR 经皮神经电刺激 OR 耳穴 OR 埋线 OR 腕踝针 OR 艾灸)<br>CBM:<br>(Advance search)<br>#1: "针刺"[常用字段:智能] OR "针灸"[常用字段:智能] OR "电针"[常用字段:智能] OR "温针"[常用字段:智能] OR "揿针"[常用字段:智能] OR "经皮神经电刺激"[常用字段:智能] OR "耳穴"[常用字段:智能] OR "埋线"[常用字段:智能] OR "腕踝针"[常用字段:智能]<br>#2: "骨癌痛"[常用字段:智能] OR "癌性骨痛"[常用字段:智能] OR "骨转移痛"[常用字段:智能] OR "骨癌疼痛"[常用字段:智能] OR "骨转移"[常用字段:智能] OR "骨肉瘤"[常用字段:智能] OR "骨癌"[常用字段:智能] OR "软骨肉瘤"[常用字段:智能] OR "恶性骨肿瘤"[常用字段:智能]<br>#3: #1 AND #2	3

Section and Topic	Item #	Checklist item	Location where item is reported
		Pubmed:<br>(Advance search)<br>#1 ((((((((cancer-induced bone pain[MeSH Terms]) OR (bone cancer pain[MeSH Terms])) OR (bone metastasis pain[MeSH Terms])) OR (bone cancer[MeSH Terms])) OR (bone metastasis[MeSH Terms])) OR (Osteosarcoma[MeSH Terms])) OR (Bone neoplasm[MeSH Terms])) OR (cancer of the bone[MeSH Terms]))<br>#2 ((((((((((acupuncture[MeSH Terms]) OR (electroacupuncture[MeSH Terms])) OR (manual acupuncture[MeSH Terms])) OR (moxibustion[MeSH Terms])) OR (catgut-embedding therapy[MeSH Terms])) OR (transcutaneous electrical acupoint stimulation[MeSH Terms])) OR (auricular point[MeSH Terms])) OR (thumb-tack acupuncture[MeSH Terms])) OR (wrist-ankle acupuncture[MeSH Terms])) OR (warm acupuncture[MeSH Terms]))<br>#3=#1 AND #2<br>Embase:<br>(Advance search)<br>#1 ‘cancer-induced bone pain’:ab,ti OR ‘bone cancer pain’:ab,ti OR ‘bone metastasis pain’:ab,ti OR ‘bone cancer’:ab,ti OR ‘bone metastasis’:ab,ti OR ‘Osteosarcoma’:ab,ti OR ‘Bone neoplasm’:ab,ti OR ‘cancer of the bone’:ab,ti<br>#2 ‘acupuncture’:ab,ti OR ‘electroacupuncture’:ab,ti OR ‘manual acupuncture’:ab,ti OR ‘moxibustion’:ab,ti OR ‘catgut-embedding therapy’:ab,ti OR ‘transcutaneous electrical acupoint stimulation’:ab,ti OR ‘auricular point’:ab,ti OR ‘thumb-tack acupuncture’:ab,ti OR ‘wrist-ankle acupuncture’:ab,ti OR ‘warm acupuncture’:ab,ti (41,645)<br>#3=#1 AND #2<br><br>Cochrane<br>(Advance search)<br>#1 (cancer-induced bone pain):ab,ti,kw OR (bone cancer pain):ab,ti,kw OR (bone metastasis pain):ab,ti,kw OR (bone cancer):ab,ti,kw OR (bone metastasis):ab,ti,kw OR (Osteosarcoma):ab,ti,kw OR (Bone neoplasm):ab,ti,kw OR (cancer of the bone):ab,ti,kw0<br>#2 (acupuncture):ab,ti,kw OR (electroacupuncture):ab,ti,kw OR (manual acupuncture):ab,ti,kw OR (moxibustion):ab,ti,kw OR (catgut-embedding therapy):ab,ti,kw OR (transcutaneous electrical acupoint stimulation):ab,ti,kw OR (auricular point):ab,ti,kw OR (thumb-tack acupuncture):ab,ti,kw OR (wrist-ankle acupuncture):ab,ti,kw OR (warm acupuncture):ab,ti,kw<br>#3=#1 AND #2	
Selection process	8	Specify the methods used to decide whether a study met the inclusion criteria of the review, including how many reviewers screened each record and each report retrieved, whether they worked independently, and if applicable, details of automation tools used in the process.<br><br>Two reviewers (Zhao-bo Yan and Zhi-miao MuRong) independently searched the articles using the abovementioned search terms and examined all studies independently according to the inclusion criteria. EndNote software was used to manage citations obtained through the database search. The extracted data were cross-checked, and discrepancies were resolved by discussion and mediated by a third reviewer (Mai-lan Liu).<br><br>The detail was shown in the following flowchart.	3

Section and Topic	Item #	Checklist item	Location where item is reported
		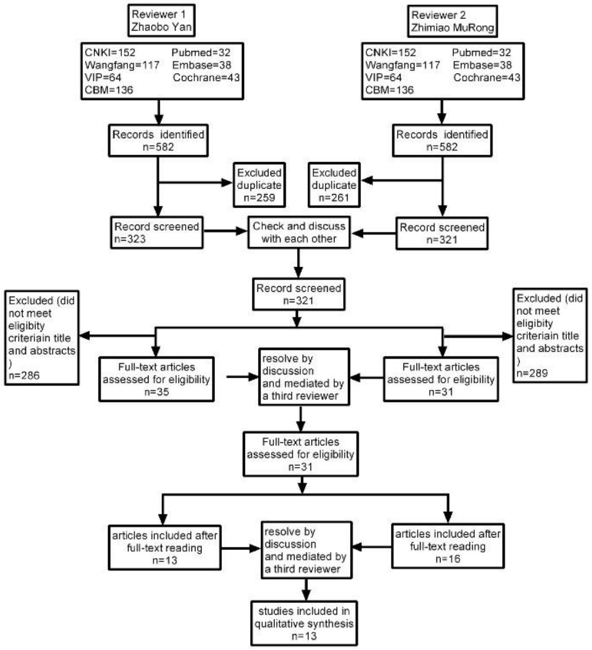	
Data collection process	9	Specify the methods used to collect data from reports, including how many reviewers collected data from each report, whether they worked independently, any processes for obtaining or confirming data from study investigators, and if applicable, details of automation tools used in the process.<br><br>Two reviewers (Zhao-bo Yan and Zhi-miao MuRong) collected data from included studies independently. We designed a data-extraction form using Microsoft Excel 2016 (https://kdocs.cn/l/clR5XTPPUfPa) that was used to extract key information from the articles. The key information will be extracted from studies that meet the inclusion criteria for data analysis. The extracted data mainly included the first author, publication year, country, sample size, age, cancer type, therapy types, control group types, outcomes, duration of intervention, and acupoints. If the above information was unclear, the report's corresponding authors were contacted to provide clarification or additional detail. The extracted data were cross-checked, and discrepancies were resolved by discussion and mediated by a third reviewer (Mai-lan Liu).	4

Section and Topic	Item #	Checklist item	Location where item is reported
Data items	10a	List and define all outcomes for which data were sought. Specify whether all results that were compatible with each outcome domain in each study were sought (e.g. for all measures, time points, analyses), and if not, the methods used to decide which results to collect.<br><br>We extracted following outcome measures assessing efficacy of acupuncture in relieving CIBP:<br>Primary outcome: pain intensity<br>Reference outcomes: pain relief rate, frequency of breakthrough pain, analgesic onset time, analgesia duration, quality of life.<br>pain intensity was described as the primary outcome, which should be reported in each included study. Pain intensity can be measured by a pain measurement, such as the Visual Analog Scale for Pain (VAS Pain), the Numeric Rating Scale for Pain (NRS Pain), or the item of "the current pain item" in the Brief Pain Inventory (BPI).<br><br>We determined pain relief rate, frequency of breakthrough pain, analgesic onset time, analgesia duration, quality of life, and adverse events as reference outcomes. Eight studies focused on quality of life, and the methods used for quality of life varied widely. Among eight studies, and three studies used the European Organization for Research and Treatment of Cancer Quality of Life Questionnaire Core 30(EORTCQLQ-C30), three studies chose the Karnofsky Performance Status Scale (KPS). In addition, the Eastern Cooperative Oncology Group (ECOG) and the Prostate Cancer-Specific Quality of Life Instrument (PROSQOLI) were used in the remaining two studies, respectively. If data were available in a study at multiple time points, we only extracted data at the latest possible time point of each period.	4
	10b	List and define all other variables for which data were sought (e.g. participant and intervention characteristics, funding sources). Describe any assumptions made about any missing or unclear information.<br>We collected data on:<br>the report: author, year, and source of publication;<br>the study: sample characteristics, social demography, and definition and criteria used for CIBP;<br>the participants: cancer types (primary bone cancer or other types of primary cancer companied by bone metastases), survival expectations (≥3 months), diagnostic (by imaging or biopsy), clinical manifestation (there was well-defined localized pain in CIBP patients).<br>the research design and features: sampling mechanism, treatment assignment mechanism, adherence;<br>the intervention: type, duration, frequency, acupoints.	4
Study risk of bias assessment	11	Specify the methods used to assess risk of bias in the included studies, including details of the tool(s) used, how many reviewers assessed each study and whether they worked independently, and if applicable, details of automation tools used in the process.<br>Evaluation of risk of bias: included studies' quality was appraised with the Cochrane collaboration tool in 7 terms, which included random sequence generation, allocation concealment, blinding of participants and personnel, blinding of outcome assessment, incomplete outcome data, selective reporting, and other bias. Each term would be rated as low risk, high risk and unclear. The evaluation was conducted independently by two reviewers (Zhao-bo Yan and Zhi-miao MuRong); any disagreements would be resolved by a third reviewer (Mai-lan Liu).	4

Section and Topic	Item #	Checklist item	Location where item is reported
		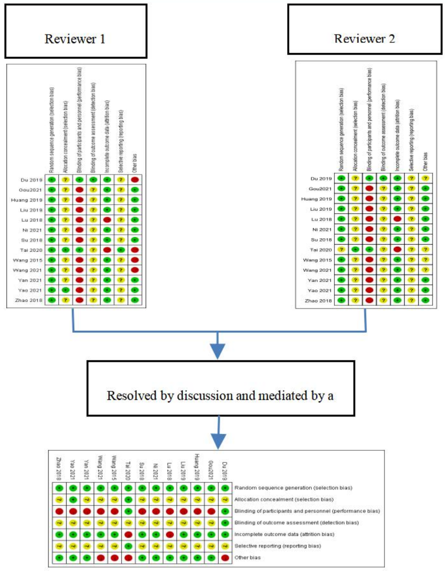	
Effect measures	12	Specify for each outcome the effect measure(s) (e.g. risk ratio, mean difference) used in the synthesis or presentation of results.<br><br>The pain relief rate belongs to dichotomous outcomes, hence, a risk ratio (RR) meta-analysis was used to pool the data from pain relief rate.<br>Pain intensity, frequency of breakthrough pain, analgesic onset time, and analgesia duration were observed in the same way across studies. We used mean difference (MD) effect sizes (Cohen's d) and their 95% confidence intervals (CIs) to pool the data of the abovementioned outcomes in the present study. The included studies used different measurement scales to assess the quality of life, and there was a large variation in the evaluation methods across the scales; pooling data from different scales was not always appropriate. However, we performed a meta-analysis based on data from three studies using the EORTC QLQ-C30 scale and the other three using the KPS scale, and we used mean difference (MD) effect sizes (Cohen's d) and their 95% confidence intervals (CIs) to calculate the data from these outcomes.	4
Synthesis methods	13a	Describe the processes used to decide which studies were eligible for each synthesis (e.g. tabulating the study intervention characteristics and comparing against the planned groups for each synthesis (item #5)).<br><br>We attempted to categorize the included interventions along two dimensions: (1) acupuncture plus control treatment vs. control treatment; (2) acupuncture alone vs. control treatment.<br>However, only two studies in our meta-analysis evaluated the effects of acupuncture alone, and they only provide information about pain intensity. Therefore, we only performed a categorical analysis on the pain intensity.	5

<table>
<tr><th>Section and Topic</th><th>Item #</th><th>Checklist item</th><th>Location where item is reported</th></tr>
<tr><td></td><td>13b</td><td>Describe any methods required to prepare the data for presentation or synthesis, such as handling of missing summary statistics, or data conversions.

The studies we included present their outcomes as Mean ± Standard deviation (Mean ±SD), so we did not convert any reported results.</td><td>/</td></tr>
<tr><td></td><td>13c</td><td>Describe any methods used to tabulate or visually display results of individual studies and syntheses.
We use Review Manager Software (5.3 version) to make forest maps and the risk of bias assessment map.</td><td>4</td></tr>
<tr><td></td><td>13d</td><td>Describe any methods used to synthesize results and provide a rationale for the choice(s). If meta-analysis was performed, describe the model(s), method(s) to identify the presence and extent of statistical heterogeneity, and software package(s) used.
We performed meta-analyses for outcomes that included two or more studies. The effect size of dichotomous and continuous data was presented as relative risk (RR) and mean difference (MD); both were reported with a 95% confidence interval. The heterogeneity among trials was identified by the $\chi 2$ test and quantified by the I2 statistic. When heterogeneity test was acceptable (P > 0.1, $I2$ ≤ 50%), a fixed-effects model was used for meta-analysis. When the heterogeneity was significant (P ≤ 0.1, $I2$ > 50%), a random-effects model was used for meta-analysis. The meta-analysis was performed using Review Manager Software (5.3 version).</td><td>4</td></tr>
</table>

outcome	Counts	Model(s)	methods	I^2	Q	Software
Pain intensity (Acupuncture plus control treatment vs control treatment)	11	MD	Random-effects	98%	<0.1	RMS (5.3)
Pain intensity (Acupuncture alone vs control treatment)	2	MD	Random-effects	95%	<0.1	RMS (5.3)
Pain relife rate	8	RR	Fixed-effects	43%	=0.09	RMS (5.3)
Analgesic onset time	2	MD	Random-effects	89%	=0.003	RMS (5.3)
Analgesia duration	2	MD	Random-effects	93%	<0.1	RMS (5.3)
Global health status (quality of life)	3	MD	Fixed-effects	0%	=0.66	RMS (5.3)
Physical functioning (quality of life)	3	MD	Random-effects	92%	<0.1	RMS (5.3)
Pain (quality of life)	2	MD	Random-effects	99%	<0.1	RMS (5.3)
Emotional (quality of life)	2	MD	Random-effects	56%	=0.13	RMS (5.3)
Cognitive (quality of life)	2	MD	Random-effects	97%	<0.1	RMS (5.3)
KPS(quality of life)	3	MD	Random-effects	72%	=0.13	RMS (5.3)

Section and Topic	Item #	Checklist item	Location where item is reported
	13e	Describe any methods used to explore possible causes of heterogeneity among study results (e.g. subgroup analysis, meta-regression).<br>We used subgroup analysis to explore possible causes of heterogeneity among study results.	4
	13f	Describe any sensitivity analyses conducted to assess robustness of the synthesized results.<br>We did not conduct any sensitivity analyses in our study.	/
Reporting bias assessment	14	Describe any methods used to assess risk of bias due to missing results in a synthesis (arising from reporting biases).<br>Because of the limited number of trails included for each comparison in the meta-analysis, funnel plots were not feasible. Therefore, we could not fully evaluate publication bias.	/
Certainty assessment	15	Describe any methods used to assess certainty (or confidence) in the body of evidence for an outcome.<br>We used the five GRADE considerations (study limitations, consistency of effect, imprecision, indirectness, and publication bias) to assess the certainty of the body of evidence as it related to the studies that contributed data to the meta-analyses for the prespecified outcomes. We assessed the certainty of evidence as high, moderate, low, or very low. We used GRADEpro GDT software to prepare the 'Summary of findings' tables (GRADEpro 3.6version).	4
**RESULTS**			

<table>
<tr><th>Section and Topic</th><th>Item #</th><th>Checklist item</th><th>Location where item is reported</th></tr>
<tr><td>Study selection</td><td>16a</td><td>Describe the results of the search and selection process, from the number of records identified in the search to the number of studies included in the review, ideally using a flow diagram.

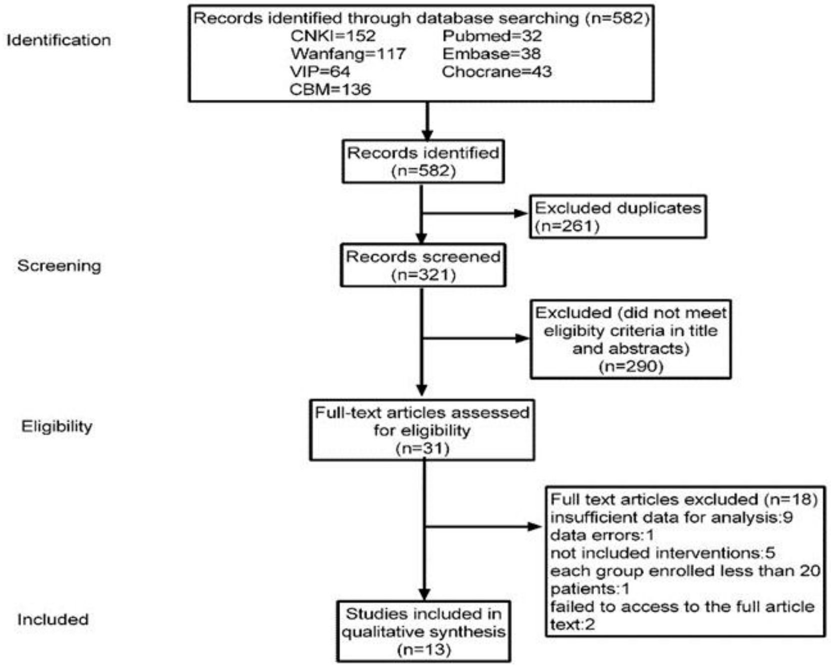

</td><td>5</td></tr>
<tr><td></td><td>16b</td><td>Cite studies that might appear to meet the inclusion criteria, but which were excluded, and explain why they were excluded.

1.CH. Y, W. L, Y. M, W. X, L. Y, W. L. Clinical Study of Wrist-Ankle Acupuncture Combined with Eight Methods of Intelligent Turtle in the Treatment of Moderate Pain with Bone Metastases from Lung Cancer [in Chinese]. Journal of Chinese Medicine (2020) 35(Z2):145.
Reason for deletion: due to missing pain score.
2. Reason for deletion: due to data errors.
3. Jie W. Clinical Study of Warm Acupuncture in the Treatment of Moderate to Severe Pain with Bone Metastases from Lung Cancer [Master's Thesis]. Guangdong China: Guangzhou University of Chinese Medicine (2019).
Reason for deletion: the subjects in this study were treated with Xiang-Sha-Liu-Jun-zi decoction, which is considered an unacceptable intervention.
4. Johnson JS, M;Wu, JS;Carlson, LE. A Feasibility Trial of Mindfulness Meditation and Acupuncture for the Treatment of Bone Pain in Women with Metastatic Cancer (Mindmap). Psychosomatic medicine (2015) 77(3):A88 - A9.
Reason for deletion: each group enrolled less than 20 patients.
5. Sima L, Yin C. Efficacy of Electroacupuncture for Bone Metastatic Cancer Patients with Neuropathic Pain: A Randomized Controlled</td><td>5</td></tr>
</table>

Section and Topic	Item #	Checklist item	Location where item is reported
		Trial. Journal of clinical oncology (2009) 27(15):9534.<br>Reason for deletion: failed to access to the full article.	
Study characteristics	17	Table 2 shows the characteristics of the included clinical trials	5/19
Risk of bias in studies	18	Present assessments of risk of bias for each included study.<br><br>Regarding selection bias, all studies mentioned the randomization method using a random number table with a low risk of bias. Only two studies provided information about allocation concealment via the use of sealed envelopes and were low risk of allocation concealment. However, allocation concealment was unclear in the remaining studies. Acupuncture research is difficult to implement a blinding design in clinical practice owing to the special nature of acupuncture. Only two studies reported the blinding of patients to be rated at low risk of performance bias. Additional studies did not mention blinding design or sham acupuncture treatment and should be considered the open-label study to be rated at high risk. One study mentioned the blinding of assessors and was rated at low risk of detection bias, additional studies were unclear about this domain. We assessed two studies as having a high risk of attrition bias for dropout rates, and attrition bias were low in the other 11 studies. Only one study was registered in the Chinese Clinical Trial Registry; we obtained the evidence that there was no reporting bias from this study scheme and rated it at low risk. Additional studies were unclear about this domain. Four included studies had other biases due to the following reasons: two studies did not describe clearly the age range of the patients, and another two studies did not provide the information about the treatment duration. A summary of the risk of bias in each of the included trials is presented in Figure 2.<br><br>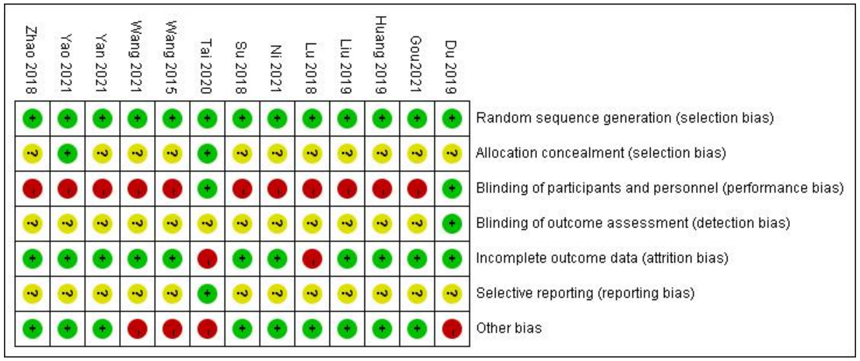	6

Section and Topic	Item #	Checklist item	Location where item is reported
Results of individual studies	19	For all outcomes, present, for each study: (a) summary statistics for each group (where appropriate) and (b) an effect estimate and its precision (e.g. confidence/credible interval), ideally using structured tables or plots.<br><br>For an example of individual study results presented for a continuous outcome, see “Fig 3. Meta-analysis of comparative effects between acupuncture plus control treatment and control treatment for cancer patients with CIBP, on reduction in pain score.	5~6
Results of syntheses	20a	For each synthesis, briefly summarise the characteristics and risk of bias among contributing studies.<br>We included 13 studies in the final qualitative analysis, all published between 2015 and 2021. A total of 1069 patients were included, with 536 in the experimental group and 533 in the control group. The largest sample size was 144 cases in a study by Yao, while the smallest sample size was 48 cases in the study by Du. The participants ranged from 18 to 84 years and included patients who were all diagnosed with bone metastases. Seven kinds of acupuncture techniques were applied across included studies, including wrist-ankle acupuncture (WAA), thumb-tack acupuncture (TTA), auricular point acupressure (APA), manual acupuncture (MA), warm acupuncture (WA), transcutaneous electrical acupoint stimulation (TEAS), and catgut-embedding therapy (CET). Control treatments in control groups comprised opioids, zoledronic acid, and nerve block. The needle retention time, the number of sessions, duration, and acupuncture point varied with acupuncture types. Eleven studies compared acupuncture plus control treatment with control treatment, one study compared wrist-ankle acupuncture treatment alone with analgesic therapy, and the remaining one compared TEAS with the combination of analgesic therapy and sham TEAS. Pain intensity as a primary outcome was used to evaluate the effect of acupuncture; VAS and NRS were the most frequently used measurement tools for pain intensity. Five studies reported VAS scores, seven studies reported NRS scores, in addition one study appraised the pain intensity by BPI scores. In terms of reference outcomes, eight studies reported a pain relief rate, one study measured the changes in the frequency of breakthrough pain, and two studies measure the analgesic onset time and analgesia duration after acupuncture treatments. Eight studies focused on quality of life, and the methods used for quality of life varied widely. Among eight studies, and three studies used the European Organization for Research and Treatment of Cancer Quality of Life Questionnaire Core 30(EORTCQLQ-C30), three studies chose the Karnofsky Performance Status Scale (KPS). In addition, the Eastern Cooperative Oncology Group (ECOG) and the Prostate Cancer-Specific Quality of Life Instrument (PROSQOLI) were used in the remaining two studies, respectively. Four studies reported adverse events. Notably, seven kinds of acupuncture treatments had been used across 13 included studies, complicating this system review's analyses. The change in the acupuncture technique brings a corresponding change in the treatment frequency, duration of treatment, needle retention time, number of sessions, and needling depth, all of which might have been potential biasing factors.	5~6
	20b	Present results of all statistical syntheses conducted. If meta-analysis was done, present for each the summary estimate and its precision (e.g. confidence/credible interval) and measures of statistical heterogeneity. If comparing groups, describe the direction of the effect.	6~7

Section and Topic	Item #	Checklist item	Location where item is reported
		“Pain intensity (VAS/RNS/BPI, 0 to 10 scale; Hight score indicates severe pain intensity): Eleven studies (862 participants) compared acupuncture plus control treatment with control treatment, the pooled results from above studies showed a marked beneficial effect of acupuncture, however, considerable heterogeneity was existed (MD = -1.34, 95% CI -1.74 to -0.94; Q<0.1; I2 = 98%, P<0.01).	

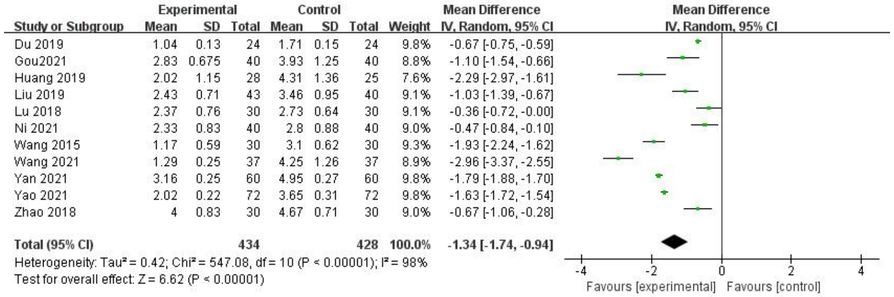

Section and Topic	Item #	Checklist item	Location where item is reported
	20c	Present results of all investigations of possible causes of heterogeneity among study results.	6

Pain intensity		study	Experimental total	Control total	I²/Q	Mean difference (95% CI)	*P*
Subgroup analysis of acupoints type	Ear points	2(CM)	58	55	0%/0.35	-1.99 [-2.27, -1.71]	＜0.01
	wrist-ankle acupoints	2(DH)	80	77	98%/＜0.1	-1.99 [-3.88, -0.10]	=0.04
	meridian acupoints	6(ABEIJK)	256	256	99%/＜0.1	-1.05 [-1.56, -0.54]	＜0.01
	combination	1(F)	40	40	-	-0.47 [-0.84, -0.10]	0.01
Subgroup analysis of	zoledronic acid	2(CK)	58	55	94%/＜0.1	-1.45 [-3.04, 0.13]	＜0.01

In our study, we only analyzed the source of heterogeneity in pain intensity (acupuncture plus control treatment vs control treatment) because this meta-analysis included 11 clinical studies, and the subgroup analysis showed that acupoint types partly explained the heterogeneity among studies.

Section and Topic	Item #	Checklist item	Location where item is reported

control group	analgesic drugs	8(ABDEFHIM)	304	301	98%/<0.1	-1.45 [-3.04, 0.13]	0.004
	nerve block	1(J)	72	72	-	-1.63 [-1.72, -1.54]	0.07
Subgroup analysis of program length	n≤2weeks	4(BEFI)	170	170	97%/<0.1	-0.94 [-1.78, -0.09]	0.07
	4weeks≥n>2weeks	3(ADJ)	139	136	99%/<0.1	-1.11 [-1.86, -0.36]	<0.01
	8weeks≥n>4weeks	2(CK)	58	55	94%/<0.1	-1.45 [-3.04, 0.13]	<0.01

A: Du 2019; B: Gou 2021; C: Huang 2019; D: Liu 2019; E: Lu 2018; F: Ni 2021; G: Su 2018; H: Wang 2021; I: Yan 2021; J: Yao 2021; K: Zhao 2018; L: Tai 2020; M: Wang 2015.

Section and Topic	Item #	Checklist item	Location where item is reported
	20d	Present results of all sensitivity analyses conducted to assess the robustness of the synthesized results.<br>We did not perform a sensitivity analysis.	/
Reporting biases	21	Present assessments of risk of bias due to missing results (arising from reporting biases) for each synthesis assessed.<br>Because of the limited number of trails included for each comparison in the meta-analysis, funnel plots were not feasible. Therefore, we could not fully evaluate publication bias.	/
Certainty of evidence	22	Present assessments of certainty (or confidence) in the body of evidence for each outcome assessed.<br>The GRADE system was used to assess the quality of evidence for the outcomes in this review. In the present study, every outcome was qualified as “very low”. From the perspective of methodological quality criteria, most included studies failed to perform effective blinding or allocation concealment, which may result in a risk of bias. Inconsistency might exist because different methods measured the outcomes, and several acupuncture techniques were used across included studies. Some meta-analyses included a low number of studies which added the imprecision of evidence. Considerable heterogeneity existing in most meta-analyses was associated with the increase in inconsistency.	8
**DISCUSSION**			
Discussion	23a	Provide a general interpretation of the results in the context of other evidence.<br>Thirteen RCTs (with 1069 patients) were included, and all studies were at high risk of bias owing to lack of blinding or other bias. Eleven studies evaluated the effectiveness of acupuncture as a complementary therapy, and showed that acupuncture plus control treatment (compared with control treatment) was connected with reduced pain intensity (MD = -1.34, 95% CI -1.74 to -0.94; Q<0.1; I2 = 98%, P<0.01). Subgroup analyses based on acupoints type partly explain the potential heterogeneity. The results also showed that acupuncture plus control treatment (compared with control treatment) was connected with relieving pain intensity, increasing the pain relief rate, reducing the frequency of breakthrough pain, shortening analgesic onset time, extending the analgesic duration, and improving the quality of life. We have no sufficient evidence to prove the effectiveness of acupuncture alone. Four RCTs reported only adverse events related to opioids’ side effects. Evidence was qualified as “very low” because of low methodological quality, considerable heterogeneity or a low number of included studies.	8~9
	23b	Discuss any limitations of the evidence included in the review.<br>This study has several limitations. First of all, there is a certain inclusion bias in this study. On the one hand, the literature search was limited to articles published in Chinese or English, excluding studies published in other languages such as Japanese, Korean, and German; on the other hand, in most cases, positive trials are published rather than negative ones, however, negative findings had important implications for our research. We had not retrieved studies with negative results, more avenues should be tried in the future, looking for negative reports.<br>Secondly, CIBP is the most common type of cancer pain. The subjects included in current review were all cancer patients with bone metastases, which always indicates that patients presented an advanced tumor stage. Primary cancer may also be a source of cancer pain at this stage. Cancer pain other than CIBP may have significantly influenced study outcomes. However, some studies included in this review do not explicitly state that pain in cancer patients only includes CIBP. Future clinical studies should strictly design inclusion and exclusion criteria to reduce the influence of irrelevant factors on the results.<br>Thirdly, the studies included in this review were not of high methodology quality. According to the seven risks of bias domains of the	10~11

Section and Topic	Item #	Checklist item	Location where item is reported
		Cochrane collaboration tool, no studies were assessed as having a low risk of bias across all domains. Only two studies described the method of allocation concealment; 10 studies lacked blinding design; two reported dropout events; only one was registered in the Chinese Clinical Trial Registry; we obtained evidence that there was no reporting bias in this study from the research scheme. However, we were not sure about other studies. The above bias interfered with the validity and reliability of the outcome to a varying degree and resulted in a very low quality of evidence. We strongly suggest that researchers should provide a reasonable design of randomization, allocation concealment, and blinding for future RCTs.<br><br>Lastly, all 13 studies included a relatively small sample size (n = 48-144), which was likely to reduce the outcomes' precision and produce misleading results. Based on the analysis above, future RCTs should include greater numbers of participants, be registered on relevant online platforms, disclose necessary information to improve the transparency and authenticity of clinical trials and facilitate the provision of reference information for scientific researchers.	
	23c	Discuss any limitations of the review processes used.<br>We acknowledge several limitations…Although we intend to make a comprehensive evaluation of the effectiveness of acupuncture on CIBP, not only assessing the pain score and pain relief rate and across the evolution of the frequency of breakthrough pain, analgesic onset time, and analgesic duration，and our findings demonstrated that acupuncture might have advantages in reducing the frequency of breakthrough pain, shortening analgesic onset time, and extending the analgesic duration. However, it should be noted that corresponding meta-analyses included a small number of RCTs (n=2) and the effect of acupuncture as adjunctive therapy may have been overestimated, and the overall quality of evidence was rated as very low. In addition, because of the limited number of trails included for each comparison in the meta-analysis, funnel plots were not feasible. Therefore, we could not fully evaluate publication bias.	10~11
	23d	Discuss implications of the results for practice, policy, and future research.<br>Firstly, in the future, attempts should be made to include RCTs in multiple languages to reduce the impact of inclusion bias on study results.<br>Secondly, future clinical studies on acupuncture in treating bone cancer pain should set strict inclusion and exclusion criteria to ensure that the included subjects only suffer from CIBP and are not affected by other cancer pain.<br>Thirdly, we strongly suggest that researchers should provide a reasonable design of randomization, allocation concealment, and blinding for future RCTs.<br>Lastly, future RCTs should include greater numbers of participants, be registered on relevant online platforms, disclose necessary information to improve the transparency and authenticity of clinical trials and facilitate the provision of reference information for scientific researchers.	10~11
**OTHER INFORMATION**			
Registration and protocol	24a	No registration.	/
	24b	No protocol.	/
	24c	Describe and explain any amendments to information provided at registration or in the protocol.<br><br>No registration.	/
Support	25	Describe sources of financial or non-financial support for the review, and the role of the funders or sponsors in the review.	12

Section and Topic	Item #	Checklist item	Location where item is reported
		This study was supported by grants from the National Natural Science Foundation of China (Grant Nos. 82074559), Key Scientific Research Project of Hunan Education Department (Grant Nos. 21A0235), Hunan Science and Technology Talents Project (Grant Nos. 2019TJ-Q04), Hunan Province "Lotus Scholar Award Program" (Xiang Jiao Tong [2020] 58.), Training Program for Excellent Young Innovators of Changsha (Grant Nos. kq1905036).	
Competing interests	26	Declare any competing interests of review authors.<br><br>No competing interests.	11
Availability of data, code and other materials	27	Report which of the following are publicly available and where they can be found: template data collection forms; data extracted from included studies; data used for all analyses; analytic code; any other materials used in the review.<br><br>The original contributions presented in the study are included in the article/Supplementary Material; further inquiries can be directed to the corresponding authors.	11

Table 1. Details of the PRISMA checklist

Database	The search strategy	Counts

CNKI	TKA=('骨癌痛'+'癌性骨痛'+'骨转移痛'+'骨癌疼痛'+'骨转移'+'骨肉瘤'+'骨癌'+'软骨肉瘤'+'恶性骨肿瘤')*('针刺'+'针灸'+'电针'+'温针'+'揿针'+'经皮神经电刺激'+'耳穴'+'埋线'+'腕踝针'+'艾灸')	152
Wanfang	题名或关键词: ("骨癌痛" or "癌性骨痛" or "骨转移痛" or "骨癌疼痛" or "骨转移" or "骨肉瘤" or "骨癌" or "软骨肉瘤" or "恶性骨肿瘤") and 题名或关键词:("针刺" or "针灸" or "电针" or "温针" or "揿针" or "经皮神经电刺激" or "耳穴" or "埋线" or "腕踝针" or "艾灸")	117
VIP	M=(骨癌痛 OR 癌性骨痛 OR 骨转移痛 OR 骨癌疼痛 OR 骨转移 OR 骨肉瘤 OR 骨癌 OR 软骨肉瘤 OR 恶性骨肿瘤) AND M=(针刺 OR 针灸 OR 电针 OR 温针 OR 揿针 OR 经皮神经电刺激 OR 耳穴 OR 埋线 OR 腕踝针 OR 艾灸)	64
CBM	"骨癌痛"[常用字段:智能] OR "癌性骨痛"[常用字段:智能] OR "骨转移痛"[常用字段:智能] OR "骨癌疼痛"[常用字段:智能] OR "骨转移"[常用字段:智能] OR "骨肉瘤"[常用字段:智能] OR "骨癌"[常用字段:智能] OR "软骨肉瘤"[常用字段:智能] OR "恶性骨肿瘤"[常用字段:智能]	136
Pubmed	#1 ((((((((cancer-induced bone pain[MeSH Terms]) OR (bone cancer pain[MeSH Terms])) OR (bone metastasis pain[MeSH Terms])) OR (bone cancer[MeSH Terms])) OR (bone metastasis[MeSH Terms])) OR (Osteosarcoma[MeSH Terms])) OR (Bone neoplasm[MeSH Terms])) OR (cancer of the bone[MeSH Terms]))<br>#2 ((((((((((acupuncture[MeSH Terms]) OR (electroacupuncture[MeSH Terms])) OR (manual acupuncture[MeSH Terms])) OR (moxibustion[MeSH Terms])) OR (catgut-embedding therapy[MeSH Terms])) OR (transcutaneous electrical acupoint stimulation[MeSH Terms])) OR (auricular point[MeSH Terms])) OR (thumb-tack acupuncture[MeSH Terms])) OR (wrist-ankle acupuncture[MeSH Terms])) OR (warm acupuncture[MeSH Terms]))<br>#3=#1 AND #2	32
Embase	#1 'cancer-induced bone pain':ab,ti OR 'bone cancer pain':ab,ti OR 'bone metastasis pain':ab,ti OR 'bone cancer':ab,ti OR 'bone metastasis':ab,ti OR 'Osteosarcoma':ab,ti OR 'Bone neoplasm':ab,ti OR 'cancer of the bone':ab,ti<br>#2 ' acupuncture ' :ab,ti OR ' electroacupuncture ' :ab,ti OR ' manual acupuncture ' :ab,ti OR ' moxibustion ' :ab,ti OR ' catgut-embedding therapy ' :ab,ti OR ' transcutaneous electrical acupoint stimulation ' :ab,ti OR ' auricular point ' :ab,ti OR ' thumb-tack acupuncture ' :ab,ti OR ' wrist-ankle acupuncture':ab,ti OR 'warm acupuncture':ab,ti (41,645)<br>#3=#1 AND #2	38

Cochrane	#1 (cancer-induced bone pain):ab,ti,kw OR (bone cancer pain):ab,ti,kw OR (bone metastasis pain):ab,ti,kw OR (bone cancer):ab,ti,kw OR (bone metastasis):ab,ti,kw OR (Osteosarcoma):ab,ti,kw OR (Bone neoplasm):ab,ti,kw OR (cancer of the bone):ab,ti,kw<br>#2 (acupuncture):ab,ti,kw OR (electroacupuncture):ab,ti,kw OR (manual acupuncture):ab,ti,kw OR (moxibustion):ab,ti,kw OR (catgut-embedding therapy):ab,ti,kw OR (transcutaneous electrical acupoint stimulation):ab,ti,kw OR (auricular point):ab,ti,kw OR (thumb-tack acupuncture):ab,ti,kw OR (wrist-ankle acupuncture):ab,ti,kw OR (warm acupuncture):ab,ti,kw<br>#3=#1 AND #2	43

Table 2. Details of the specific research strategy corresponding to each database
